# “Kind of blurry”: Deciphering clues to prevent, investigate and manage prescribing cascades

**DOI:** 10.1371/journal.pone.0272418

**Published:** 2022-08-31

**Authors:** Barbara Farrell, Emily Galley, Lianne Jeffs, Pam Howell, Lisa M. McCarthy

**Affiliations:** 1 Bruyère Research Institute, Ottawa, Ontario, Canada; 2 Department of Family Medicine, University of Ottawa, Ottawa, Ontario, Canada; 3 School of Pharmacy, University of Waterloo, Waterloo, Ontario, Canada; 4 Lunenfeld-Tanenbaum Research Institute, Sinai Health, Toronto, Ontario, Canada; 5 Institute of Health Policy, Management and Evaluation, University of Toronto, Toronto, Ontario, Canada; 6 Institute for Better Health, Trillium Health Partners, Mississauga, Ontario, Canada; 7 Leslie Dan Faculty of Pharmacy and Department of Family and Community Medicine, University of Toronto, Toronto, Ontario, Canada; 8 Women’s College Research Institute, Toronto, Ontario, Canada; Universidad de Santiago de Chile Facultad de Ciencias Medicas, CHILE

## Abstract

**Background:**

Prescribing cascades, where a medication is used to treat the side effect of another medication, contribute to polypharmacy and related morbidity. Little is known about clinicians’ and patients’ experiences with prescribing cascades. In this study, we explored why and how prescribing cascades occur across a variety of care settings and how they are managed.

**Methods and findings:**

This descriptive qualitative study employed semi-structured interviews with older adults who may have experienced a prescribing cascade(s), their caregivers, and healthcare providers. Interviewees were recruited through physician referral from a Geriatric Day Hospital, two long-term care homes in Ottawa, Ontario, and through self-referral across Ontario, Canada. An inductive approach was used to code data and determine themes. Thirty-one interviews were conducted for ten unique patient cases. Some interviewees were involved in more than one case, resulting in 22 unique interviewees. Three themes were identified. First, recognition of prescribing cascades is linked to awareness of medication side effects. Second, investigation and management of prescribing cascades is simultaneous and iterative (rather than linear and sequential). Third, prevention of prescribing cascades requires intentional strategies to help people anticipate and recognize medication side effects. Difficulty with recruitment from both long-term care homes and through self-referral was the central limitation. This exemplifies challenges associated with studying a poorly recognized and underexplored phenomenon.

**Conclusions:**

In order to better recognize, investigate and manage prescribing cascades, clinicians and patients need to know more about medication side effects; they need to ask ‘can this be caused by a drug?’ when signs and symptoms arise or worsen; and they need access to information about medication experiences to have benefit-risk discussions and make decisions about deprescribing. Approaches for raising public awareness of prescribing cascades should be trialed to raise the profile of this issue and facilitate continued exploration of the phenomenon.

## Introduction

Prescribing cascades, where a medication is used to treat the side effect of another medication, contribute to polypharmacy and related morbidity, problems that are of particular interest to those who care for older adults [1, 2]. They can occur when medication-related side effects are attributed to a new medical condition [2]. Many prescribing cascades have been identified and their potential impact on patients described [3–9]. Much of the existing research on prescribing cascades has used healthcare administrative data to assess their prevalence and impact [10–16]. Resources have been identified that can help people prevent, detect and reverse prescribing cascades but there remain outstanding knowledge gaps regarding both clinicians and patients’ experiences with identifying and addressing them as well as how and why prescribing cascades occur in practice [9, 17, 18].

In a previous study, we interviewed older adults attending a Geriatric Day Hospital program (i.e., a referral-based ambulatory clinic with an interprofessional care team) who may have experienced one or more prescribing cascades and their healthcare providers to understand how cascades develop and how they are resolved [19]. We found that prescribing cascades were difficult to identify and that their development was impacted by varying levels of awareness of medications, their side effects and related cascades, patients struggling to report their medication experiences, and of personal accountability regarding decisions about medication changes. We also found that having access to an interdisciplinary team environment and relevant information about medication use and effects were important enablers for identifying cascades and resolving them.

In this study, we build upon this earlier work by drawing from a wider range of practice environments and practitioners. One goal was to interview older adults who may have better recall of their medication experiences than patients recruited to the first study. Through qualitative interviews with patients, and then their healthcare team members and family caregivers, we explored why and how prescribing cascades occur, how they impact patients and how they are managed. This approach allowed us to begin our inquiry with the individuals experiencing prescribing cascades and then explore the perspectives of those involved in prescribing and/or managing their prescribing cascades.

## Methods

### Design

This descriptive qualitative study [20–22] included conducting and analyzing semi-structured one-on-one interviews with patients, their family caregivers, and healthcare providers that were involved in an assessment of possible prescribing cascades. The COREQ criteria were used to guide this report [23] (S1 Checklist).

### Setting

Interviewees were recruited through physician referral from a Geriatric Day Hospital (GDH) program, two long-term care (LTC) homes in Ottawa, Ontario, and through self-referral across Ontario, Canada.

### Researcher characteristics

The three investigators have a health care background (two PharmD–BF and LMM, and one RN/PhD—LJ); all three are affiliated with different research organizations. A pharmacist research assistant (BScPhm)–PH, conducted the majority of interviews, and a Masters-trained research associate, EG, with expertise in qualitative research supported data analysis. One pharmacist investigator, BF, also works clinically in the GDH program and only conducted interviews with research participants associated with the other recruitment sites. She and the research associate coded transcripts; all investigators reviewed code summaries and were involved in determining themes. All team members were female.

### Participants, purposeful sampling and consent process

**Fig 1:**
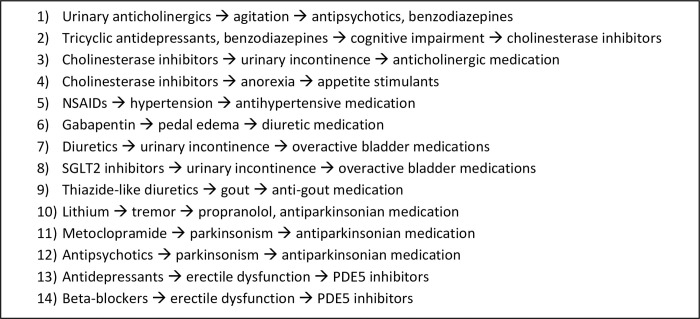
What are examples of prescribing cascades?

Patient participants (>65 years old; able to complete an interview in English) were identified using two approaches. First, for those from the GDH program and those in the two LTC homes, on-site physicians and pharmacists identified and approached people who may have experienced one or more prescribing cascades about their interest in the study. Site presentations and recruitment posters provided recruiting clinicians with a list of possible prescribing cascades (Fig 1). If interest was confirmed, the clinician provided the research team with a completed screening form specifying the suspected prescribing cascade(s) and providing contact information for the person. The research team first contacted the person by phone and then obtained written consent in a face-to-face appointment. Second, we offered a self-referral option for members of the public. These participants were recruited through advertising across Ontario using social media advertisements (Twitter, Facebook); newsletter announcements; and posters in pharmacies, family practice clinics, local community centres, retirement homes, and libraries. Electronic advertisements were distributed through email list servs of the RTOERO (https://rtoero.ca/about/), an organization representing >80 000 retired education workers (sample advertisement, S1 Appendix). Self-referring individuals were contacted by phone and oral consent was obtained and documented as per REB protocol.

Following the consent and interview process, patient participants were asked to provide contact information for and permission to contact a caregiver and/or healthcare provider(s) who could provide additional information about the prescribing cascade experience. These individuals were contacted separately by hand-delivered invitations (e.g., to pharmacies or family practices), by registered mail or by phone for consent (with signed consent form returned by mail or email).

The study was approved by the Bruyère Research Ethics Board (REB) (protocol #M16-17-009) and the University of Toronto Research Ethics Board (#36481).

### Data collection

Interviews were conducted between July and December 2019 by the pharmacist research assistant (PH) and one pharmacist investigator (BF). Interviewees could opt for in person or telephone interviews. Only the participant and interviewer were present; there were no repeat interviews. Interview guides (patient, caregiver, healthcare provider versions, S2 Appendix) adapted from the pilot interviews [19], focused on factors associated with the development, identification and resolution of their suspected prescribing cascade(s); the resulting clinical impact; and participant insights regarding potential interventions to prevent, identify or resolve prescribing cascades. Interviewers completed memos following each interview detailing overall impressions and recommendations for questions of the other interviewees involved in the case. All interviews were audio recorded and transcribed verbatim. Transcripts were de-identified and verified for accuracy against recordings by the research associate, then paired with interviewer memos.

### Data analysis

After the first three interviews had been transcribed (August 2019), the transcripts were distributed to the research team for familiarization, an important preliminary step in qualitative analysis when data collection and coding are fulfilled by different members of the research team [24, 25]. In our case, multi-disciplinary research team members were affiliated with different institutions in two cities, thus, early familiarization, and sharing of observational notes at monthly teleconference meetings, supported a coherent, collaborative analysis and introduced an element of reflexivity to the process [26]. Familiarization was on-going throughout the analysis process and all members of the research team participated regardless of whether they also participated in coding.

Initial coding of the first three transcripts was conducted by two members of the research team (the research associate–EG, and one pharmacist investigator—BF) in August 2019. Both analysts independently applied an inductive coding strategy. Codes and supporting quotations were compared and discussed during a full team meeting during which proposed codes were reviewed for inter-coder and source data agreement; codes were correspondingly accepted, altered, merged, divided, or dropped. Based on this review, a working code book was created and the first three transcripts re-coded by two analysts; they met to discuss coding agreement and code clarity and refined the codebook by clustering codes and insights into categories (August-September 2019). The same two members of the research team subsequently completed all coding.

Coding was on-going with completed transcripts distributed to the analysts in batches approximately every two weeks (August–December 2019); new transcripts were divided between the two coders so only one coder was responsible for a given transcript. Every two weeks, coders reviewed one of the other coder’s completed transcripts to ensure consistency of code application and inhibit coder deviation from the established code book [27]; verifying agreement on coding served to improve the rigour of the coding process [26]. Following this review, the coders met to discuss any issues, ideas, or concerns, e.g. emergent or weak codes, disagreements in application of codes, and the codebook was updated accordingly with older codebooks being retained for verification purposes.

Transcript analysis was conducted using NVivo 12 [28], allowing for the disaggregation and sorting of coded data and facilitating inter-coder consistency and transparency. Following the completion of the coding process, node and sub-node summaries were reviewed by the full research team during a face-to-face analysis retreat (January 2020) to identify patterns, categories, and themes [29]. Coding summaries were completed, noting overlaps, redundancies, and potential inter-node relationships, and subsequently verified by the two coders, who then compiled a final analysis report for review by the team.

An audit trail includes raw data (recordings, transcripts, memos), codebook versions, coded transcripts, meeting minutes and reports filed by date.

## Results

Thirty-one interviews were conducted for ten unique cases: six GDH patients, two LTC residents, two self-referrals; four caregivers, three pharmacists and five physicians were interviewed with some pharmacists and physicians interviewed about more than one case, resulting in 22 unique interviewees. Interview duration ranged from 14 to 65 minutes (mean 54 minutes). Hereafter, patients, residents and self-referred members of the public are referred to as ‘patients’.

In Table 1, we describe the patient demographics, the prescribing cascades prompting study referral and provide short case summaries. Many patients experienced more than one potential cascade and/or were experiencing an adverse effect that could be caused by more than one of their medications. Often potential cascades could not be confirmed due to lack of information regarding medication’s reasons for use or chronology of medication prescribing. In other situations, different or additional prescribing cascades were identified through discussion with different members of the healthcare team. Many patients recruited from the GDH and LTC were uncertain about the impact of potential cascades on their health when asked. Across cases, it is evident that while people experienced suffering from side effects, it often took time for medication causes to be identified as such and for a plan to manage them to be formulated. One participant, from the self-referral stream shared her experience about the impact of medication adverse effects on her function and quality of life. She experienced chronic diarrhea for many years, which was likely caused by medications because it resolved after medications known to cause diarrhea were stopped:

*“I would go to the bathroom and my bowels would run like I was peeing…*. *I could hardly go anywhere*. *I was a music teacher and I was also an art teacher*. *I gave up teaching art because I couldn’t be there without finding a bathroom all the time*. *The music*, *at first*, *I tried to sit in the front row where I was the lead alto*, *so I would sit there*. *I played my music for forty years*. *It’s my passion*. *And I had to actually quit that*.*”* [P108]

**Table 1:** Case summaries of prescribing cascade experiences.

Case (sex; age)	Potential cascade(s) listed by physician as prompting referral for the study, or self-referral	Interview sources	Case summary
F; 69	• fentanyl → sweating → oxybutynin • fentanyl → nausea → dimenhydrinate • fentanyl → tremors, anxiety → lorazepam	Patient (P101) GDH physician (MD406) GDH Pharmacist (PH407) Community Pharmacist (PH404)	In this case, multiple medications might have been contributing to symptoms like sweating, tremors, anxiety and nausea; it was difficult to attribute symptoms to one medication. The patient reported initial significant analgesic benefit from fentanyl but became increasingly concerned about side effects over time. The patient agreed to reduce the doses of some medications (e.g., fentanyl, escitalopram, lorazepam) but this did not improve all symptoms and a small dose of oxybutynin was retained for the sweating. Nausea was improved with the use of ginger, allowing the dimenhydrinate to be stopped. Additional prescribing cascades were identified by the GDH pharmacist: prednisone → osteoporosis risk → bisphosphonate; celecoxib → ulcer risk → pantoprazole, dimenhydrinate/oxybutynin → dry mouth → artificial saliva spray.
F; 69	• trazodone, dimenhydrinate, baclofen, cetirizine → urinary retention → furosemide • pantoprazole → low magnesium → magnesium supplement	Patient (P102) GDH physician (MD406) GDH Pharmacist (PH407) Family physician (MD408)	Multiple medications might have contributed to urinary retention and cognitive impairment. The patient was initially open to dose reduction of medications affecting cognition (e.g., trazodone, baclofen, dimenhydrinate) but not to changes to diclofenac, furosemide or magnesium, which she perceived to be of significant benefit for pain, passing urine and fibromyalgia, respectively. Additional prescribing cascades were identified by the GDH pharmacist: diclofenac → ulcer risk → pantoprazole, diclofenac → edema → furosemide, caffeine → insomnia → trazodone. Trazodone was tapered and ultimately stopped, being successfully replaced with melatonin and a sleep mask. Caffeine use continued.
F; 70	• rosuvastatin → muscle pain → diclofenac, amitriptyline, hydromorphone, ibuprofen • diclofenac, ibuprofen → GI symptoms → pantoprazole, ranitidine • pantoprazole → low B12 → B12 supplement	Patient (P103) Caregiver (CG303) GDH pharmacist (PH407) GDH physician (MD409)	This patient was very unsure about reasons for use of medications, did not make any connections between symptoms and medications, and was unsure about reasons medications had been changed in the past. She had a documented history of having had stopped celecoxib due to hypertension. After assessment, it was determined her pain was related to osteoarthritis, not rosuvastatin. Plans to reduce medications to determine if other medications could be stopped were made but not yet implemented at the time of the interview. Additional prescribing cascades were identified by the GDH pharmacist: high dose venlafaxine and regular use of pseudoephedrine → hypertension → two antihypertensives; amitriptyline/diphenhydramine → urinary retention → mirabegron.
M; 84	• venlafaxine → hypertension → amlodipine • amlodipine → edema → furosemide	Patient (P104) Caregiver (CG306) GDH physician (MD412) GDH pharmacist (PH407)	In this case, the prescribing cascades listed by the referring clinician were not confirmed. Hypertension preceded the venlafaxine and was thought not to be made worse by the low dose. Both the patient and caregiver felt there was benefit from the venlafaxine and did not want to stop. No attempt to reduce the amlodipine or change to a different antihypertensive was made and furosemide was felt to be indicated for this patient given history of aortic stenosis, as ankle swelling had worsened in the past with furosemide dose reduction.
F; 85	• metformin, domperidone → diarrhea → loperamide • amlodipine → edema → furosemide	Patient (P105) GDH physician (MD412) GDH pharmacist (PH407)	This patient had had longstanding diarrhea with several potential medication contributors to severity (including pantoprazole, high doses of omega-3 fatty acids, caffeine). Metformin and pantoprazole were stopped during the GDH admission with improvement in the diarrhea. There was some reluctance to stop domperidone as the original reason for use (i.e., nausea or gastroparesis) was unclear. Amlodipine dose had just been reduced and no changes made to the furosemide at the time of the interview. The GDH pharmacist identified an additional possible prescribing cascade: omega-3 fatty acids → antihyperglycemics.
F; 73	Self-referred	Patient (P107) Caregiver (CG309)	This patient self-referred to the study because she felt that several of the medications she takes for her rheumatoid arthritis (celecoxib, methotrexate, hydroxychloroquine) were causing stomach upset for which she takes pantoprazole. She was concerned about potential side effects of pantoprazole but found her two physicians (family doctor and gastroenterologist) disagreed about whether she should continue it. She said she struggles with knowing the medications provide benefit but necessitate an additional drug to manage their side effects.
F; 82	Self-referred	Patient (P108) Caregiver (CG 308) Family Health Team Pharmacist (PH411)	This patient self-referred to the study because of her history with multiple side effects and prescribing cascades significantly impacting her quality of life. The patient, caregiver and pharmacist confirmed a seven year timeline of events that began with a doubling of metformin and rosuvastatin along with the addition of gliclazide which led to a decreased in blood glucose prompting cessation of gliclazide and initiation of sitagliptin. This was followed by the onset of severe diarrhea, initially diagnosed as irritable bowel syndrome and managed with loperamide, which continued for some years until a gastroenterologist eventually asked her to stop metformin and sitagliptin (after which insulin was started). Diarrhea resolved and loperamide was stopped. At the time of the initial diabetes medication changes (when metformin was doubled and sitagliptin added), she also developed atrial fibrillation which was investigated by several cardiologists who eventually advised that she had no heart problems. Ultimately the atrial fibrillation was thought to be drug-induced brought on by medications; this also improved with the cessation of sitagliptin and metformin. Concurrently, she had severe leg pain which was treated in the emergency room with tramadol which caused severe nausea and shaking. Pain was subsequently felt to be due to rosuvastatin which was stopped by a rheumatologist with resolution of pain. A benzodiazepine was started at some point (reason for use suspected to be insomnia or anxiety but not confirmed) and the patient fell sustaining physical injury and subsequent rapid decline, after which the benzodiazepine was stopped. Though she was next prescribed duloxetine for her mood, she did not take it as she was worried about more potential medication adverse effects. At roughly this time, the patient read about the association of benzodiazepines and falls, and the possibility of prescribing cascades. She also described an experience where an increase in hydrochlorothiazide and furosemide resulted in an increase in blood sugar with need for higher doses of diabetes medications (which improved when the furosemide was stopped and hydrochlorothiazide returned to its original dose). Through these experiences and her own research, the patient has now become a vocal advocate for the need for public awareness of polypharmacy.
F; 74	• pregabalin, amlodipine → edema → furosemide • methylphenidate → high heart rate and blood pressure → metoprolol, irbesartan	Patient (P109) GDH pharmacist (PH407) GDH physician (MD 409)	In this case, it was challenging to confirm prescribing cascades due to lack of information about reasons for use of some medications (e.g., furosemide) and unclear chronology (i.e., uncertainty whether furosemide started before or after pregabalin/amlodipine). The patient was not interested in most dose reductions making it difficult to investigate or manage prescribing cascades. She described excellent pain relief from pregabalin and feeling of energy from methylphenidate and these benefits outweighed impact of any potential side effects for her. Fluoxetine was stopped as mood was good (hoping this would also improve fatigue which might have led to the prescription for methylphenidate). Amlodipine may have been contributing to edema but was not changed during the admission. The GDH pharmacist identified additional potential prescribing cascades: methotrexate → folic acid supplement; combination of acetylsalicylic acid/clopidogrel/fluoxetine → ulcer risk → pantoprazole; furosemide → potassium loss → potassium supplement; the patient was also taking prednisone and so the question of whether a bisphosphonate should be added arose.
F; 86	• hydromorphone contin → constipation → polyethylene glycol • amlodipine → edema → furosemide	Resident (R201) LTC physician (MD401)	The resident appeared willing to accept side effects of her pain medication due to its effectiveness. From her physician, we learned she was prescribed hydromorphone for severe, acute pain and the patient said she found this effective. Though reluctant to take laxatives initially, polyethylene glycol, psyllium fibre and senna were managing constipation so well now that the patient told us she was not having side effects from the hydromorphone. Of the two cascades, the physician felt she would likely have more success reducing the amlodipine and then tapering furosemide (which was initially started for an acute pleural effusion but no longer needed for that reason and not effective for the ankle swelling patient was now having).
F; 91	• candesartan/ hydrochlorothiazide → increased blood glucose → metformin	Resident (R202) LTC physician (MD401)	In this case, the resident began taking candesartan/hydrochlorothiazide combination which she recalls resulted in an increase in blood sugar. This appears to have led to either the addition of, or an increase in metformin dose several weeks later (difficult to discern from electronic records; patient can’t recall order of medications clearly; new physician in LTC doesn’t have past records)). After the hydrochlorothiazide component was stopped, blood sugar fell and metformin was reduced.

Abbreviations: M Male, F Female, GDH Geriatric Day Hospital, LTC Long-Term Care, PH pharmacist, MD physician, CG Caregiver

**Fig 2:**
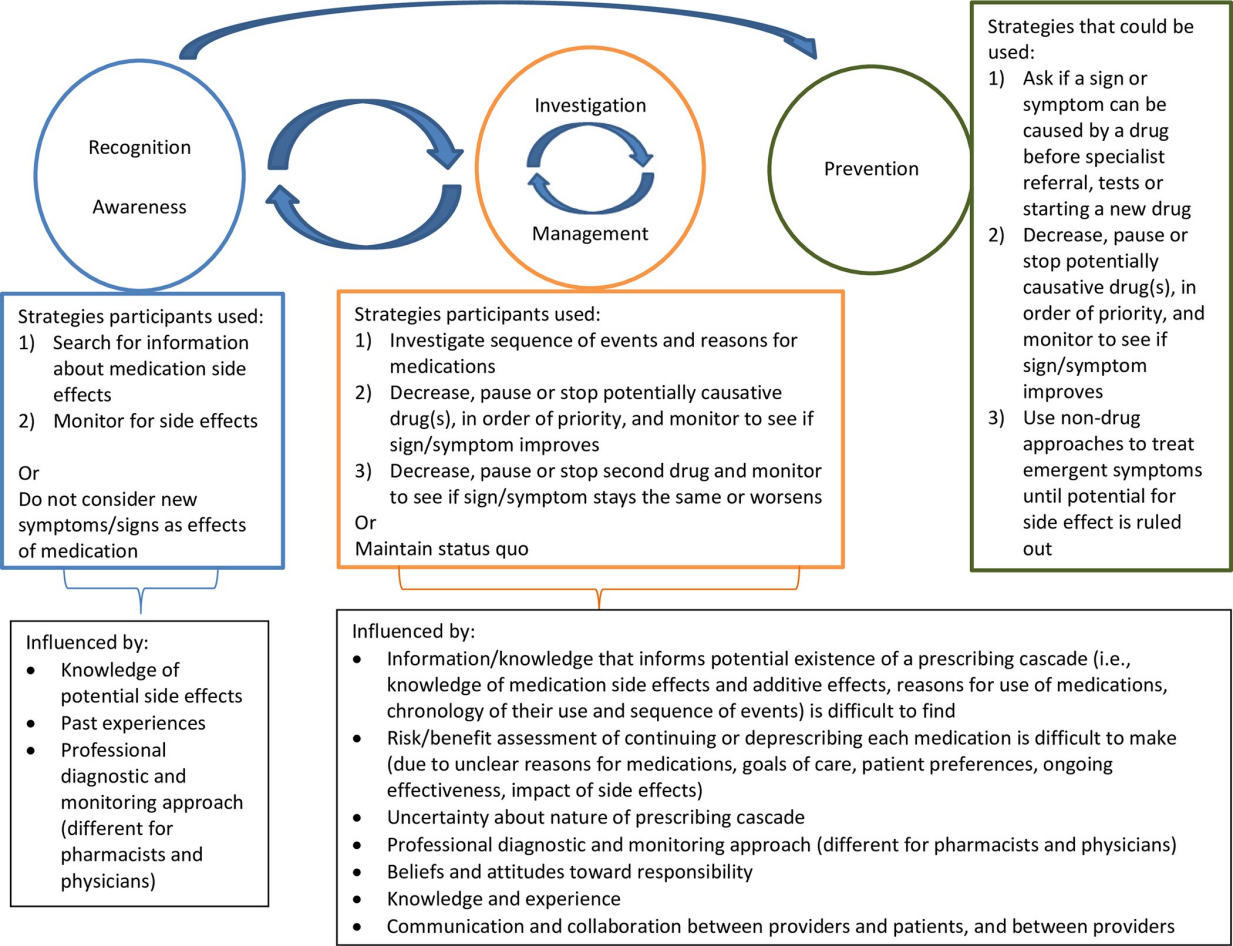
Themes related to recognition, investigation, management and prevention of prescribing cascades.

Three central themes were identified related to prescribing cascade recognition, prevention, investigation and potential management strategies. For each, we describe the strategies that patients, caregivers and healthcare providers used and factors that influenced each. Fig 2 illustrates the intersecting, bi-directional and iterative relationships amongst the themes.

### 1. Recognition of prescribing cascades is linked to awareness of medication side effects

While participating patients usually said they understood that a medication could be used to treat the side effect of another medication, they had not typically heard the term ‘prescribing cascade’ and did not apply it to their own medication experiences. Most patients and caregivers demonstrated a low level of knowledge and understanding of medications, usually recalling only a very general reason for the medication’s use and approximate duration. They reported they had not received information about side effects or, if they received information, it was not easily understandable, resulting in poor knowledge of potential side effects.

While many patients expressed frustration about this lack of knowledge about medications and their side effects and an interest in knowing more, few articulated strategies for gathering such information. Many tended to trust their physician to know how a medication might affect them:

*“I believed right from childhood that the doctor*, *he knows more than me*. *So… I blindly took what the doctor prescribed me”*. [P104]

However, they still expressed uncertainty at how knowledgeable physicians might be regarding medications, pointing to the narrow scope of some physician practice (e.g. specialists) and to their reliance on pharmacists to *“figure it out”*. [P104] Patients who related the highest amount of understanding of their medications described significant personal efforts to find information, including conducting independent research, as they sought alternatives to perceived issues and actively engaged with their healthcare providers, particularly their pharmacists.

Amongst physicians, awareness and knowledge of specific prescribing cascades was more prevalent but they did not describe a common approach to successfully identifying them in practice. The ability to recognize specific cascades was influenced by physicians’ training, practice experience and exposure to prescribing cascades:

*“I work in an environment where we have patients with a significant degree of polypharmacy and I’ve worked there for some years now so I think it’s just generally on my radar and also on my background in care of the elderly means that I have a significant degree of training around polypharmacy and deprescribing”*. [P103-MD409]

One physician stated that through involvement in this study, they were more aware of examples of prescribing cascades and this made it easier for them to recognize them:

*“Up until the last year*, *I would have never even equated elevated blood sugar with hydrochlorothiazide”*. [R202-MD401]

Physicians and pharmacists suggested that aspects of their formal education subsequently influenced their different approaches to assessing new symptoms and ability to recognize potential medication side effects and thereby potential prescribing cascades. Medical education was described as a disease-focused approach to symptom management:

*“It’s not kind of in the rubric of the physician having not been formally trained in pharmacology*, *for example*, *to sort of immediately question*, *oh*, *could this symptom be caused by a medication*. *So*, *it’s more common I think and more usual for the physician to say*, *oh*, *what does this symptom represent in terms of a new medical condition and can I treat this*?*”* [P101-MD406]

Pharmacy education was described as being aligned with a medication-focused approach with emphasis on linking symptoms to potential medication risks. These divergent educational paradigms were suggested as a source of physicians’ self-reported challenge in recognizing emergent signs and symptoms as potential side effects associated with causative drugs.

Healthcare providers relied on patients reporting emergent symptoms and indicating a relationship to a new medication; when this did not occur, they were less likely to investigate the possibility of side effects and more likely to treat a new sign or symptom as an emerging medical condition. The likelihood of recognizing symptoms as potential medication-related side effects decreased over time i.e., health care providers were less likely to consider that new side effects can arise from long-term medications:

*“I would say at the initial onset*, *absolutely*, *we would be paying closer attention as to whether we were seeing side effects*. *But once it’s been onboard for quite some time*, *it kind of escapes the mind and probably doesn’t come up as frequently*.*”* [R201-MD401]

Overall, an awareness of the potential for side effects of medications, knowledge about the types of side effects that medications can cause, and an intentional monitoring approach is needed by both patients and their healthcare providers to as a starting point for recognizing, and then investigating and managing prescribing cascades.

### 2. Investigation and management of prescribing cascades is simultaneous and iterative

The experiences of our interviewees demonstrated that investigation and management of prescribing cascades are not linear or discrete processes. These processes are inextricably linked, often occurring simultaneously. Significant knowledge about medication use and trialing periods of deprescribing (i.e., reducing doses or stopping a medication that might be causing a side effect) are needed to confirm hypotheses about causation.

To investigate whether a prescribing cascade has occurred, healthcare providers described starting with the *“time-consuming”* [P102-MD408] process of gathering a history of medication use. This included identifying the sequence of events (i.e., timing of medications and appearance of signs/symptoms) and the reasons particular medications are being used or were prescribed. To accomplish this, they spoke about needing to use detective work, find clues and *“decipher”* [R201-MD401] the information uncovered:

*“People have been on things for years or they’re not really sure why they’re taking something… you’re not even sure who the original prescriber was… you’re kind of teasing information together based on consults you can get your hands on and whatever comes from the GP”*. [R201-MD401]

Challenges included limited, or lost, history in both paper and electronic charts, patient’s difficulty in recalling information (particularly when experiencing cognitive impairment) and uncertainty arising when medications have multiple possible reasons for use:

*“People probably did have prescribing cascades*, *but we don’t know the timeline or we don’t understand the chronology of how drugs were prescribed to be able to say with any degree of certainty that this in fact [happened]*.*”* [R201-MD401]

Further uncertainty about the original reason for a medication hampered clinicians’ ability to assess its effectiveness. This made it difficult to assess the relative risks and benefits of continuing or stopping a medication, an integral component of investigating and managing a possible prescribing cascade.

Even when the original reason a medication was prescribed was relatively clear, its’ ongoing reason(s) for use were often less clear, with one physician describing a particular case as *“kind of blurry*.*”* [R201-MD 401] Without an understanding of ongoing indications or timing of other medications, it was difficult to ascertain whether a prescribing cascade has occurred; this was further complicated when a long-standing medication could be associated with a symptom or side effect that has more recently emerged.

Healthcare providers spoke about how prescribing cascades do not necessarily follow a classic linear ‘medication causes side effect for which second medication is used’ scenario.

*“It’s hard to identify a pure prescribing cascade in the sense of saying*, *this medication was started at this point in time*, *it caused this side effect and in response to that*, *this other medication was prescribed to address the side effect*. *It takes time…to be able to say categorically that [drug B] was prescribed to attend or to address the side effect of [drug A] it’s hard to get down to that level*.*”* [P102-MD406]

Additionally, common or vague symptoms can sometimes be caused by multiple drugs making it difficult to sort out which medications were most likely contributing.

*“Which drug am I going to choose that’s causing nausea*? *It’s probably a combination of the 10 drugs that they’re on*.*”* [R201-MD401]

Not being able to stop each of the potentially contributing medications made it difficult to say a prescribing cascade had happened with certainty. Complexity was further increased when several medications could cause a constellation of different types of symptoms which might be treated by different types of other medications.

*“I grouped together the tremors*, *the sweating*, *and the nausea as a constellation of symptoms to which those five drugs might be contributing*.*”* [P101-PH407]

And, it was also hard to know if a medication was worsening a pre-existing medical condition already being treated by a drug.

*“Some people might say it’s not a prescribing cascade then because the second drug was started before the first drug*. *But the question is*, *are the drugs like trazodone*, *dimenhydrinate*, *baclofen and cetirizine*, *are they all making the urinary retention worse*? *I’m not sure how to answer that*.*”* [P102-PH407]

Physicians described their inclination to first establish a differential diagnosis of medical conditions, which typically does not include specific medication-related causes, to address and manage a particular sign or symptom. This often included referral to specialists who did not connect symptoms to medications as potential causes but carried out many tests and investigations. One patient related that:

*“Nobody ever linked the adverse drug reactions to a medication*, *period*. *What they did was they tried to figure out other things… I just went for a lot of tests*. *I saw four cardiologists in that time*.*”* [P108]

Pharmacists were more likely to question whether a medication could be causing the sign or symptom and to recommend deprescribing as both an investigation and management strategy.

When there were several potential prescribing cascades where more than one drug might be contributing to a sign or symptom, or where there multiple different prescribing cascades, there was a need to prioritize which potentially causative drugs to reduce first. This required consideration of the risks and benefits of each drug, goals of care, the possibility of needing to increase or add a different medication to achieve those goals, and the need for a step-wise approach with monitoring for resolution or worsening of symptoms. One pharmacist related the potential complexity of this situation:

*“You’ve got the pseudoephedrine*, *the venlafaxine*, *the ibuprofen and the diclofenac*, *all of which can be contributing to high blood pressure…if we could reduce the venlafaxine*, *stop the diclofenac*, *try to minimize the ibuprofen and reduce the Tylenol Sinus®*, *we could see if the blood pressure got better*. *And she was taking two drugs to treat her hypertension*.*”* [P103-PH407]

Situations in which a patient’s function seemed stable, or the risk-benefit of continuing or deprescribing a medication was unclear led to paralysis in making changes:

*“When you see someone’s function stable and not deteriorating*, *you don’t have as much of an impetus to do something about it*. *Yeah*, *like not rocking the boat*. *Everything is status quo*. *There’s a big reluctance to stop things as an inheriting physician because you’re like*, *well*, *what if it was really doing something super important*?*”* [R201-MD401]

Patients themselves were rarely able to describe the benefits of a medication or its adverse effects, and when they could, their balancing of these effects sometimes shifted over time, making it important to continually seek their input about changed preferences:

*“I didn’t like all the side effects*, *but it helps me with the pain*, *you know*? *By that time*, *I just cared about my pain*. *I didn’t care about the side effects*, *but now*, *because I thought that the sweating and all the things together that I feel like I’m dying*. *It was because of the medications*. *Then*, *I started worrying about it*. *But before*, *I didn’t*, *I just cared about the pain”*. [P101]

It was clear that it was hard to know who should be ultimately responsible for making changes:

*“No one is kind of taking responsibility to actually resolve problems that are hinted at people might argue*, *well*, *it’s not the gastroenterologist’s place to stop a diabetes medication*. *But if he’s investigating the side effect of the medication*, *then maybe it is the specialist’s place to do that*.*”* [P109-PH407]

Changing medications required conversations with, and agreement from, patients and amongst healthcare providers. Decisions were influenced by patient priorities and their feelings about how well the medication was working and the impact of side effects. Physician input was also essential in weighing benefit and risk:

*“When there are four or five drugs that could be contributing to this one symptom*, *it helps to have the physician perspective on which drugs it might be safer to decrease first because of the likelihood that they might be being effective in some way*.*”* [P101-PH407]

Conversations about these decisions were more challenging when there was cognitive impairment or limited understanding of medications, or when patients were reluctant to make changes. Gaining buy-in from patients involved being honest but not alarmist about side effects and potential risks and appealing to the idea of reducing medications:

*“When you sort of explain that some symptoms can be caused by medication and that if we take the tact of reducing meds*, *it will mean less of a pill burden and helps in a more positive response*.*”* [P101-MD406]

Overall, the processes for investigating and managing prescribing cascades are interconnected. Deprescribing potentially causative medications, as well as those treating side effects, is typically necessary to increase certainty that a prescribing cascade had occurred. Carrying out these processes is challenging as information to weigh risk/benefit of each medication is often lacking, people may not consider or know that a medication may be causing a side effect and taking action to investigate or manage a prescribing cascade requires knowledge, a willingness to be responsible and to facilitate communication amongst patients and prescribers.

### 3. Prevention of prescribing cascades requires intentional strategies to help people anticipate and recognize medication side effects

People spoke about strategies that could be used to prevent prescribing cascades. On the part of prescribers and pharmacists, this would involve knowing about the side effects of medications and common prescribing cascades; incorporating a ‘can this be caused by a drug’ process *“Could this symptom be caused by one or more of the medications that they’re taking…*?*”* [P101-MD406] into their assessment of new signs or symptoms; taking steps to reduce potentially causative drugs before ordering a test or adding another medication: *“Should we be thinking about deprescribing as a means of treating the symptom*?*”* [P101-MD406] and monitoring for the improvement in these signs or symptoms.

For medications being added to reduce the likelihood of another drug’s potential harm, an assessment of whether the initial drug’s ongoing benefit) is critical before a medication is added for prevention. For example, when deciding whether pantoprazole should be added to reduce the risk of bleeding for a patient taking multiple agents that increased bleeding risk:

*“One of the questions I had for her cardiologist is how long does she need to be on two anti-platelet agents*? *Because what we would rather do if we could is reduce her bleeding risk by stopping some of the drugs that increase bleeding risk*, *rather than giving a fourth drug to reduce the risk from the first three*.*”* [P109-MD409]

Interviewees indicated that current practice does not typically include consideration of deprescribing with one pharmacist stating:

*“it’s very common to have patients who have had multiple consultant visits to investigate a problem*. *And then we decide to take a chance on stopping a drug that we think might be causing the problem*, *and we stop the drug and the problem gets better*. *But no consultant has ever suggested that*.*”* [P103-PH407]

Computerized alerts, medication reviews, enhanced monitoring for side effects, including patients in monitoring for side effects, and thinking outside their own specialty area about symptoms that may be caused by a drug were raised as potential strategies for prevention:

*“I think in the context of maybe another prescribing cascade*, *if there is a very common-known side-effect*, *how do you alert someone to identify that side-effect before just treating the side-effect and thinking about the original drug*.*”* [R202-MD401]

However, these were seen to be potentially limited by alert fatigue: *“When you alert people to too many things*, *then you just start ignoring the alerts*.*”* [R202-MD401], lack of time and funding for medication reviews, lack of knowledge about side effects and reduced suspicion of side effects when they develop long after medications are initially prescribed:

*“Sometimes*, *people develop side-effects to treatments they have been on for years*, *way down the road*. *In my mind*, *I don’t always equate that to a drug right away*, *because in my mind I say*, *well*, *you tolerated for so long*, *you must be tolerating it*. *This can’t necessarily be related*. *But maybe in fact it actually is*.*”* [R202-MD401]

Several healthcare provider interviewees discussed central roles for patients in knowing their medications, identifying medication side effects and working with prescribers to prevent prescribing cascades

*“The idea is to empower people to understand that they’ve got a responsibility to collect and share this information”* [P101-PH407].

However, it was recognized that this strategy requires knowledge and confidence on the part of the patient to feel empowered to ask questions: one patient expressed that *“there is a huge need for consumers to know that they can ask questions”* [P108] Their caregiver agreed, stating that:

*“I still feel that a lot of people don’t understand that one of their medications could be causing their problem*, *and that it isn’t a health issue that’s causing their problem”*. [P108-CG308]

In summary, preventive strategies largely rely on the ability to anticipate and recognize symptoms as potential side effects of medications, to evaluate ongoing benefit of medications and to consider deprescribing before adding another medication.

## Discussion

When we began to study this phenomenon, consistent with existing literature [1, 9], we conceptualized addressing prescribing cascades as linear, discrete steps: identify, resolve, prevent. In our prior study with patients from the GDH program, we found that prescribing cascades were complex and contextually situated [19]. Prescribing cascades were difficult to identify and their development was impacted by varying levels of awareness of medications, their side effects and related cascades [19]. In this study, our findings further reinforce that a simple model involving a linear process does not adequately capture the nuances of the therapeutic challenges of prescribing cascades. Instead, we observed non-linear, intersecting, bi-directional and frequently simultaneous processes. Both investigation and management strategies facilitated recognition of existing and future prescribing cascades with a goal towards prevention. However, a baseline amount of awareness was required to initiate investigation and management; awareness was likely enhanced by past experiences with the process investigating and managing prescribing cascades. Significant time and cognitive effort were required to conduct these challenging processes.

Overall, strategies to facilitate the awareness and recognition of prescribing cascades included searching for information about side effects of medications and monitoring for those side effects. Barriers included a lack of knowledge about potential side effects (on the part of both prescribers and patients) and training that emphasizes a focus on medical conditions versus medication-related causes of signs and symptoms.

To improve the awareness and recognition of side effects that can be contributing to prescribing cascades, both healthcare providers and patients need to consciously ask ‘could this be caused by a drug?’ as part of an approach to diagnosis. Asking (and answering) this question about a sign, symptom or apparent new or worsening medical condition before referral to a specialist for additional investigations or adding a new drug treatment could prevent a prescribing cascade. There are calls for all healthcare professionals involved in managing medication therapy to incorporate this type of assessment as part of their clinical practice [2, 18]. Knowing what side effects could arise; that side effects can sometimes severely impact patients’ lives leading to specialist referral(s), additional investigation(s) and treatment(s); understanding that side effects could arise even after long periods of treatment; and how to regularly monitor for side effects will facilitate this process. This requires a shift in medical education particularly to incorporate the consideration of drug-related causes of signs and symptoms as part of the patient assessment process, greater emphasis on global knowledge about side effects of medications and age-related changes in medication pharmacokinetics and pharmacodynamics, and competencies for deprescribing. Research to identify the most common and clinically relevant prescribing cascades are underway and this information will support awareness efforts both of specific cascades and the phenomenon more broadly [30].

Strategies to both investigate and manage prescribing cascades build on this knowledge; requiring clinicians to determine reasons for use of medications, chronology of their use, and sequence of events. Deprescribing one or more potentially causative medications can help determine if a sign or symptom is influenced by a medication; deprescribing of a medication being used to treat said sign or symptom is the next important management component. However, decisions to carry out these deprescribing actions are plagued by uncertainty: lack of knowledge about why medications are being used, how well they are working, when they were started in relation to the appearance of other signs or symptoms or the use of other medications, whether medications have caused problems in the past and whether more than one medication could have additive effects all contribute to challenges assessing the risk-benefit of deprescribing and taking accountability to make changes.

To improve people’s ability to investigate and manage prescribing cascades, consistent with facilitators for deprescribing, it would be helpful to have documented clear reasons for original uses of medications, information about their effectiveness and an approach to long-term monitoring for adverse effects [31]. Healthcare providers involved in the patient’s care should have access to this information and patients themselves must be clearly informed and engaged in monitoring for both effectiveness and adverse effects. Such documentation and knowledge will help people make informed decisions about risk-benefit of continuing medications which ultimately informs discussions about deprescribing as part of both sign and symptom investigation, as well as management of a possible prescribing cascade. Such decisions are heavily influenced by the patient’s perception of risk-benefit of each medication and their readiness to make changes, something that may change over time and with additional information [32]. Clinicians must be flexible and persistent in engaging in such discussions as patients’ perceptions change.

Interprofessional collaboration and communication is also important to facilitate consultation about benefit-risk of each medication, and to obtain consent on dose changes. Healthcare provider training that emphasizes competencies for interprofessional collaboration in deprescribing must be incorporated into training programs and expectations for practice [30]. Investigation and management of prescribing cascades is more likely to occur when physicians have this knowledge and/or are working with a pharmacist guiding the process.

Designing studies to explore how and why prescribing cascades occur in practice has been challenging, particularly from a recruitment perspective. Cascades as a phenomenon are underexplored from a research perspective and sparsely recognized by providers and the public. Recruitment from both LTC homes and the self-referral streams resulted in fewer cases than originally desired. For example, our two LTC sites agreed that prescribing cascades were a significant problem within their institutions; yet one site did not refer any cases and two cases from the second site were referred by the same physician. During check-ins with our research team, the sites’ pharmacists and physicians reported low referral numbers due to suspecting, but being unable to confirm, that patients experienced prescribing cascades or feeling that cascades were resolved (e.g., by stopping a drug on admission) rendering patients ineligible. It is unclear whether this challenge is specific to the recruitment strategy used in these two sites or a challenge that would be encountered more broadly across the LTC sector.

Further, despite widespread efforts to recruit for our self-referral stream, only two people self-referred for interviews. In our recruitment materials, we did not use the phrase ‘prescribing cascade’ specifically, which is a different approach than that used by Bloomstone and colleagues [33]. In their study, which developed and tested education materials for people living with dementia and their caregivers about prescribing cascades, investigators used an increasingly recognized cascade, calcium channel blocker → edema → diuretic, to introduce the concept. Interestingly, they also noted that people struggled to understand the concept of prescribing cascades. This raises questions about whether optimal strategies for promoting awareness of the phenomenon with the public are needed or whether the public is not concerned with the issue. That said, there are growing calls for recognition of prescribing cascades as contributors to polypharmacy, a WHO-recognized contributor to medication-related harm [34]. Further research to understand optimal messaging about the phenomenon of prescribing cascades and their impact is urgently needed.

Our decision to focus on recruitment of older adults experiencing cascades may have contributed to some of the challenges we faced. Older adults often live with multiple chronic conditions which can result in being prescribing multiple medications, a risk factor for prescribing cascades. However, it is possible that some of our findings would differ with people of different ages. For example, younger people may be more knowledgeable about the effects of their medications, may have been more willing or able to participate and may have different experiences and insights; expanding the target population to include people across the lifespan merits consideration in future work.

Another opportunity for future research lies in furthering understanding about the clinical impact of prescribing cascades on patient function, quality of life, and health service utilization. We had aimed to study the clinical impact of prescribing cascades but our ability to do so was hampered by the minimal knowledge people generally had about medication effects. Studies have begun to explore this using healthcare administrative data. For example, Morris et al. explored the impact of a specific cascade on quality of life using United States Medical Expenditure Panel Survey data. They found lower physical functioning amongst those experiencing the cascade, suggesting a clinically meaningful decrease in health-related quality of life [4]. Qualitative and mixed-methods approaches can add rich layers of context to these experiences.

Our team’s next step toward developing interventions to help people investigate, manage, and prevent prescribing cascades is to use these interview findings to undertake a behavioural analysis, an initial step toward theory guided-intervention development using the Behaviour Change Wheel approach [35]. We will then identify target behaviours for healthcare providers and the public, their underlying behavioural drivers, relevant intervention types, policy categories, and applicable behaviour change techniques. Stakeholder input about the acceptability and feasibility of proposed interventions will be sought to support development, testing and evaluation of future interventions.

## Conclusion

We found that healthcare providers and members of the public do not consistently consider if signs, symptoms or new or worsening medical conditions could be caused by medications the patient is taking. If this is considered, they are not able to easily investigate whether a cascade is present. Both patients and providers struggle to access information to confirm the existence of a possible prescribing cascade or help them develop a plan to manage it. Further, investigation and management of prescribing cascades can be complex as the processes are often simultaneous, non-linear, intersecting and time-consuming. Lastly, healthcare providers and members of the public have difficulty assessing risks and benefits of continuing or deprescribing a medication, and therefore difficulty with strategizing plans for investigation and management of a prescribing cascade. To address these challenges, healthcare providers and members of the public need to be more knowledgeable about the side effects of medications, ask themselves whether new signs, symptoms or new or worsening medical conditions could be caused by a drug and be willing to discuss benefit-risk of medications to make decisions about deprescribing. Better communication about the reasons for medications and their effects will facilitate these processes. Strategies to promote the awareness of medication side effects and prescribing cascades will raise the profile of this important issue and ultimately facilitate continued exploration of the phenomenon to inform solutions.

## Supporting information

S1 Checklist COREQ checklist. (DOCX)

S1 Appendix Recruitment poster. (PDF)

S2 Appendix Interview guides. (DOCX)

S1 File Exported code book. (DOCX)

S2 File Coding summaries. (DOCX)

## References

[1] McCarthy LM, Visentin JD, Rochon PA. Assessing the scope and appropriateness of prescribing cascades. J Am Geriatr Soc. 2019;67: 1023–1026. doi: 10.1111/jgs.15800 30747997

[2] Rochon PA, Gurwitz JH. The prescribing cascade revisited. Lancet. 2017;389: 1778–1780. doi: 10.1016/S0140-6736(17)31188-1 28495154

[3] Morris EJ, Hollmann J, Hofer AK, Bhagwandass H, Oueini R, Adkins LE, et al. Evaluating the use of prescription sequence symmetry analysis as a pharmacovigilance tool: A scoping review. Res Social Adm Pharm. 2022;18: 3079–3093. doi: 10.1016/j.sapharm.2021.08.003 34376366

[4] Morris EJ, Brown JD, Manini TM, Vouri SM. Differences in health-related quality of life among adults with a potential dihydropyridine calcium channel blocker–loop diuretic prescribing cascade. Drugs and Aging. 2021;38: 625–632. doi: 10.1007/s40266-021-00868-0 34095980

[5] Nguyen PVQ, Spinelli C. Prescribing cascade in an elderly woman. Can Pharm J. 2016;149: 122–124. doi: 10.1177/1715163516640811 27212961 PMC4860747

[6] Caughey GE, Roughead EE, Pratt N, Shakib S, Vitry AI, Gilbert AL. Increased risk of hip fracture in the elderly associated with prochlorperazine: Is a prescribing cascade contributing? Pharmacoepidemiol Drug Saf. 2010;19: 977–982. doi: 10.1002/pds.2009 20623516

[7] Ribo A. Ertapenem-induced neuropsychiatric symptoms in an elderly patient with chronic kidney disease resulting to a prescribing cascade. J Pharmacovigil. 2014;02: 8–10. doi: 10.4172/2329-6887.1000152

[8] Nunnari P, Ceccarelli G, Ladiana N, Notaro P. Prescribing cascades and medications most frequently involved in pain therapy: A review. Eur Rev Med Pharmacol Sci. 2021;25: 1034–1041. doi: 10.26355/eurrev_202101_24673 33577059

[9] Brath H, Mehta N, Savage RD, Gill SS, Wu W, Bronskill SE, et al. What is known about preventing, detecting, and reversing prescribing cascades: A scoping review. J Am Geriatr Soc. 2018;66: 2079–2085. doi: 10.1111/jgs.15543 30335185

[10] Vouri SM, van Tuyl JS, Olsen MA, Xian H, Schootman M. An evaluation of a potential calcium channel blocker–lower-extremity edema–loop diuretic prescribing cascade. J Am Pharm Assoc. 2018;58: 534–539.e4. doi: 10.1016/j.japh.2018.06.014 30033126 PMC6424490

[11] Savage RD, Visentin JD, Bronskill SE, Wang X, Gruneir A, Giannakeas V, et al. Evaluation of a common prescribing cascade of calcium channel blockers and diuretics in older adults with hypertension. JAMA Intern Med. 2020;180: 643–651. doi: 10.1001/jamainternmed.2019.7087 32091538 PMC7042805

[12] Vouri SM, Jiang X, Manini TM, Solberg LM, Pepine C, Malone DC, et al. Magnitude of and characteristics associated with the treatment of calcium channel blocker-induced lower-extremity edema with loop diuretics. JAMA Netw Open. 2019;2: e1918425. doi: 10.1001/jamanetworkopen.2019.18425 31880802 PMC6991233

[13] Trenaman SC, Bowles SK, Kirkland S, Andrew MK. An examination of three prescribing cascades in a cohort of older adults with dementia. BMC Geriatr. 2021;21: 1–11. doi: 10.1186/s12877-021-02246-2 33964882 PMC8106136

[14] Elli C, Novella A, Nobili A, Ianes A, Pasina L. Laxative agents in nursing homes: An example of prescribing cascade. J Am Med Dir Assoc. 2021;22: 2559–2564. doi: 10.1016/j.jamda.2021.04.021 34023302

[15] Chen Y, Huang ST, Hsu TC, Peng LN, Hsiao FY, Chen LK. Detecting suspected prescribing cascades by prescription sequence symmetry analysis of nationwide real-world data. J Am Med Dir Assoc. 2022;23: 468–474.e6. doi: 10.1016/j.jamda.2021.06.035 34324873

[16] Read SH, Giannakeas V, Pop P, Bronskill SE, Herrmann N, Chen S, et al. Evidence of a gabapentinoid and diuretic prescribing cascade among older adults with lower back pain. J Am Geriatr Soc. 2021;69: 2842–2850. doi: 10.1111/jgs.17312 34118076

[17] Ponte ML, Wachs L, Wachs A, Serra HA. Prescribing cascade. A proposed new way to evaluate it. Medicina (B Aires). 2017;77: 13–16. Available from: https://pubmed.ncbi.nlm.nih.gov/28140305/ 28140305

[18] Piggott KL, Mehta N, Wong CL, Rochon PA. Using a clinical process map to identify prescribing cascades in your patient. BMJ. 2020;368. doi: 10.1136/bmj.m261 32075785

[19] Farrell BJ, Jeffs L, Irving H, McCarthy LM. Patient and provider perspectives on the development and resolution of prescribing cascades: A qualitative study. BMC Geriatr. 2020;20: 1–11. doi: 10.1186/s12877-020-01774-7 32977743 PMC7519478

[20] Percy WH, Kostere K, Kostere S. Generic qualitative research in psychology. Qual Rep. 2015;20: 76–85. doi: 10.46743/2160-3715/2015.2097

[21] Sandelowski M. Whatever happened to qualitative description? Res Nurs Health. 2000;23: 334–340. doi: 10.1002/1098-240x(200008)23:4&lt;334::aid-nur9&gt;3.0.co;2-g 10940958

[22] Caelli K, Ray L, Mill J. ‘Clear as mud’: Toward greater clarity in generic qualitative research. Int J Qual Methods. 2003;2: 1–13. doi: 10.1177/160940690300200201

[23] Tong A, Sainsbury P, Craig J. Consolidated criteria for reporting qualitative research (COREQ): A 32-item checklist for interviews and focus groups. Int J Qual Heal Care. 2007;19: 349–357. doi: 10.1093/intqhc/mzm042 17872937

[24] Braun V, Clarke V. Thematic analysis. In: Cooper H, Camic PM, Long DL, Panter AT, Rindskopf D, Sher KJ, editors. APA handbook of research methods in psychology, Vol 2: Research designs: Quantitative, qualitative, neuropsychological, and biological. 1st ed. American Psychological Association; 2012. pp. 57–71. doi: 10.1037/13620-000

[25] Gale NK, Heath G, Cameron E, Rashid S, Redwood S. Using the framework method for the analysis of qualitative data in multi-disciplinary health research. BMC Med Res Methodol. 2013;13: 1–8. doi: 10.1186/1471-2288-13-117 24047204 PMC3848812

[26] Cornish F, Gillespie A, Zittoun T. Collaborative analysis of qualitative data. In: Flick U, editor. SAGE Handb Qual Data Anal. SAGE Publications Ltd; 2014. pp. 79–93. doi: 10.4135/9781446282243.N6

[27] MacQueen KM, McLellan E, Kay K, Milstein B. Codebook development for team-based qualitative analysis. Field Methods. 1998;10: 31–36. doi: 10.1177/1525822X980100020301

[28] QSR International Pty Ltd. Qualitative Data Analysis Software | NVivo. [cited 11 Nov 2021]. Available: https://www.qsrinternational.com/nvivo-qualitative-data-analysis-software/home

[29] Braun V, Clarke V. Using thematic analysis in psychology. Qual Res Psychol. 2006;3: 77–101. doi: 10.1191/1478088706qp063oa

[30] Sternberg SA, Petrovic M, Onder G, Cherubini A, O’Mahony D, Gurwitz JH, et al. Identifying key prescribing cascades in older people (iKASCADE): A transnational initiative on drug safety through a sex and gender lens—rationale and design. Eur Geriatr Med. 2021;12: 475–483. doi: 10.1007/s41999-021-00480-w 33835427

[31] Linsky A, Zimmerman KM. Provider and system-level barriers to deprescribing: Interconnected problems and solutions. Public Policy Aging Rep. 2018;28: 129–133. doi: 10.1093/PPAR/PRY030

[32] Doherty AJ, Boland P, Reed J, Clegg AJ, Stephani A-M, Williams NH, et al. Barriers and facilitators to deprescribing in primary care: A systematic review. BJGP Open. 2020;4. doi: 10.3399/bjgpopen20X101096 32723784 PMC7465575

[33] Bloomstone S, Anzuoni K, Cocoros N, Gurwitz JH, Haynes K, Nair VP, et al. Prescribing cascades in persons with Alzheimer’s disease: Engaging patients, caregivers, and providers in a qualitative evaluation of print educational materials. Ther Adv drug Saf. 2020;11: 2042098620968310. doi: 10.1177/2042098620968310 33240479 PMC7675869

[34] Medication without harm—Global patient safety challenge on medication safety. Geneva: World Health Organization; 2017.

[35] Michie S, Atkins L, West R. The behaviour change wheel: A guide to designing interventions. London: Silverback Publishing; 2014.

