# Supplementary material for: “Kind of blurry”: Deciphering clues to prevent, investigate and manage prescribing cascades
Source: PLoS One. 2022 Aug 31;17(8):e0272418. doi: 10.1371/journal.pone.0272418 (PMC9432713; doi:10.1371/journal.pone.0272418)
Supplement: S1 Checklist — (DOCX) [file pone.0272418.s001.docx]

**Appendix 1 - Consolidated criteria for reporting qualitative studies (COREQ): 32-item checklist**

| **No** | **Item** | **Guide questions/description** | **Found on Submitted Manuscript Section** | **Comments** |
| --- | --- | --- | --- | --- |
| **Domain 1: Research team and reflexivity** | | | | |
| Personal Characteristics | | | | |
| 1. | Interviewer/facilitator | Which author/s conducted the interview or focus group? | Methods: Researcher characteristics |  |
| 2. | Credentials | What were the researcher's credentials? *E.g. PhD, MD* | Methods: Researcher characteristics |  |
| 3. | Occupation | What was their occupation at the time of the study? | Methods: Researcher characteristics |  |
| 4. | Gender | Was the researcher male or female? | Methods: Researcher characteristics |  |
| 5. | Experience and training | What experience or training did the researcher have? | Methods: Researcher characteristics |  |
| 6. | Relationship established | Was a relationship established prior to study commencement? | Methods: Researcher characteristics |  |
| 7. | Participant knowledge of the interviewer | What did the participants know about the researcher? e*.g. personal goals, reasons for doing the research* |  | Included in the patient consent form, available on request. |
| 8. | Interviewer characteristics | What characteristics were reported about the interviewer/facilitator? e.g. *Bias, assumptions, reasons and interests in the research topic* | Introduction  Methods: Researcher characteristics |  |
| **Domain 2: study design** | | | | |
| Theoretical framework | | | | |
| 9. | Methodological orientation and Theory | What methodological orientation was stated to underpin the study? *e.g. grounded theory, discourse analysis, ethnography, phenomenology, content analysis* | Methods: Design |  |
| Participant selection | | | | |
| 10. | Sampling | How were participants selected? *e.g. purposive, convenience, consecutive, snowball* | Methods: Participants, purposeful sampling, and consent process |  |
| 11. | Method of approach | How were participants approached? e*.g. face-to-face, telephone, mail, email* | Methods: Participants, purposeful sampling, and consent process |  |
| 12. | Sample size | How many participants were in the study? | Results |  |
| 13. | Non-participation | How many people refused to participate or dropped out? Reasons? |  | No-one approached for the study declined to participate or dropped out of study. One person was assigned a code (P106) after being referred, but was not approach about the study as their condition changed; all information related to this individual has been deleted. |
| Setting | | | | |
| 14. | Setting of data collection | Where was the data collected? e*.g. home, clinic, workplace* | Methods: Data collection |  |
| 15. | Presence of non-participants | Was anyone else present besides the participants and researchers? | Methods: Data collection |  |
| 16. | Description of sample | What are the important characteristics of the sample? *e.g. demographic data, date* | Results, Table 1 |  |
| Data collection | | | | |
| 17. | Interview guide | Were questions, prompts, guides provided by the authors? Was it pilot tested? | Methods: Data collection, Appendix 3 |  |
| 18. | Repeat interviews | Were repeat interviews carried out? If yes, how many? | Methods: Data collection |  |
| 19. | Audio/visual recording | Did the research use audio or visual recording to collect the data? | Methods: Data collection |  |
| 20. | Field notes | Were field notes made during and/or after the interview or focus group? | Methods: Data collection |  |
| 21. | Duration | What was the duration of the interviews or focus group? | Results |  |
| 22. | Data saturation | Was data saturation discussed? |  | Data saturation was discussed at monthly research team meetings and a decision made to stop patient recruitment once it was clear new data seemed to express what was already seen in previous data and no new codes or themes were apparent. |
| 23. | Transcripts returned | Were transcripts returned to participants for comment and/or correction? | Methods: Data collection |  |
| **Domain 3: analysis and findings** | | | | |
| Data analysis | | | | |
| 24. | Number of data coders | How many data coders coded the data? | Methods: Data analysis |  |
| 25. | Description of the coding tree | Did authors provide a description of the coding tree? | Methods: Data analysis | Coding document available on request. |
| 26. | Derivation of themes | Were themes identified in advance or derived from the data? | Methods: Data analysis |  |
| 27. | Software | What software, if applicable, was used to manage the data? | Methods: Data analysis |  |
| 28. | Participant checking | Did participants provide feedback on the findings? |  | Participants did not provide feedback. |
| Reporting | | | | |
| 29. | Quotations presented | Were participant quotations presented to illustrate the themes / findings? Was each quotation identified? e*.g. participant number* | Results |  |
| 30. | Data and findings consistent | Was there consistency between the data presented and the findings? | Results, Discussion |  |
| 31. | Clarity of major themes | Were major themes clearly presented in the findings? | Results |  |
| 32. | Clarity of minor themes | Is there a description of diverse cases or discussion of minor themes? | Results | Within each of the three central themes, strategies that people use as well as factors that influenced each are presented. One circumstance of significant impact on quality of life is described. Minor themes are clinical impacts. |
