# Supplementary material for: “Kind of blurry”: Deciphering clues to prevent, investigate and manage prescribing cascades
Source: PLoS One. 2022 Aug 31;17(8):e0272418. doi: 10.1371/journal.pone.0272418 (PMC9432713; doi:10.1371/journal.pone.0272418)
Supplement: S1 Appendix — (PDF) [file pone.0272418.s002.pdf]

ARE YOU TAKING A MEDICATION FOR THE SIDE EFFECTS OF ANOTHER?

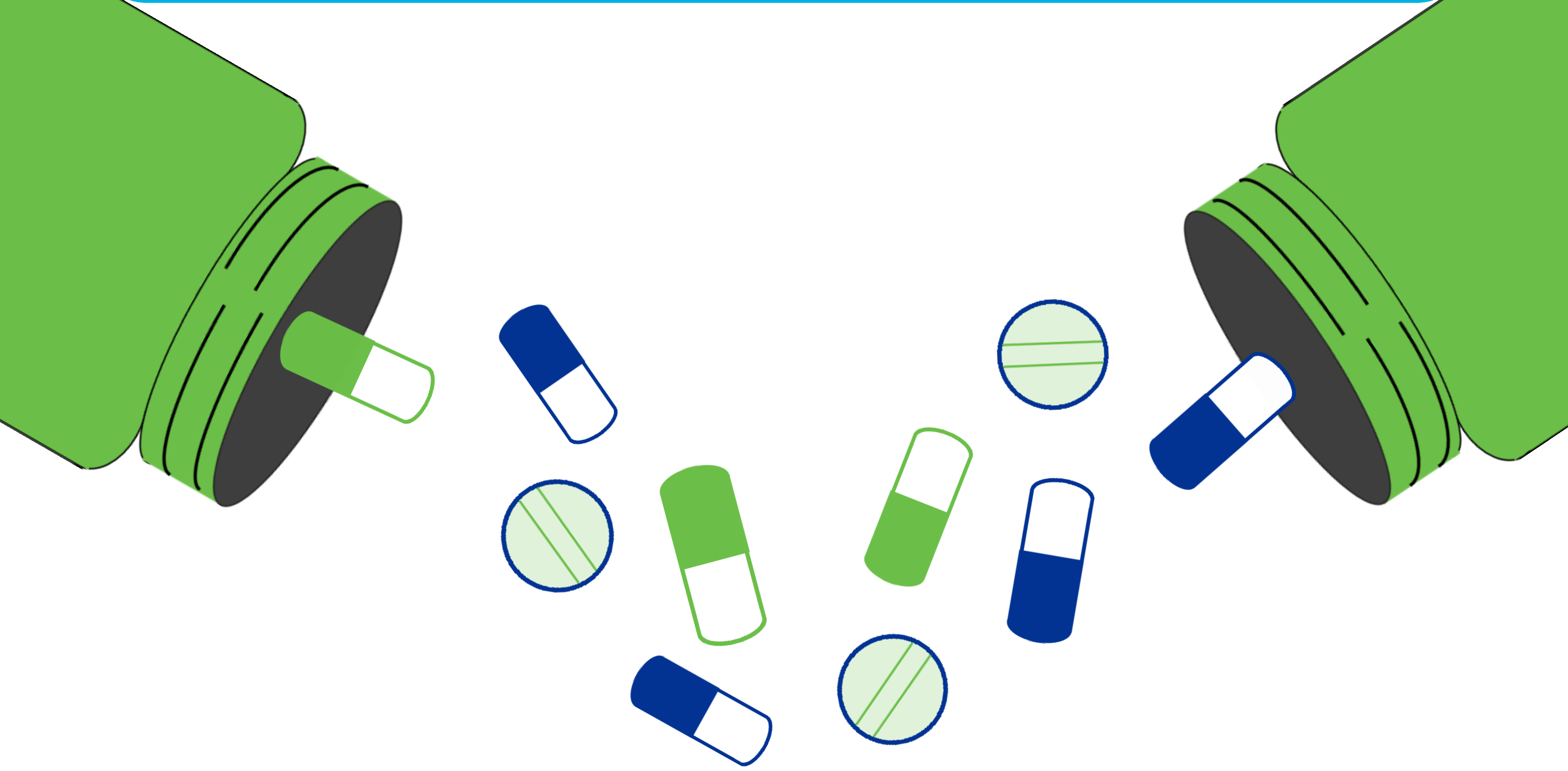

If so, we want to talk to you about your experiences.

If you're over age 65, living in Ontario and interested in learning more about or participating in this research study, please **contact us**

phone: 613-562-6262 ext. 1770

email: deprescribing@bruyere.org

INSTITUT DE RECHERCHE Bruyère RESEARCH INSTITUTE
deprescribing.org
UNIVERSITY OF TORONTO
Affilié à / Affiliated with uOttawa
