# Supplementary material for: “Kind of blurry”: Deciphering clues to prevent, investigate and manage prescribing cascades
Source: PLoS One. 2022 Aug 31;17(8):e0272418. doi: 10.1371/journal.pone.0272418 (PMC9432713; doi:10.1371/journal.pone.0272418)
Supplement: S2 Appendix — (DOCX) [file pone.0272418.s003.docx]

**Appendix 3 – Interview guides**

**Prescribing Cascades Semi-structured Patient Interview Guide**

**Preamble**

Thank you for agreeing to participate in this study. My name is <insert name> and I am a research coordinator with the Bruyère Research Institute. Our study team is hoping to hear about your experiences with your medications, more specifically about the time when you were given a medication to manage a side effect from another of your medications. I am going to ask you a series of questions but want to assure you that there are no right or wrong answers. We are interested in your thoughts and experiences. The interview will last about 30 to 60 minutes but if at any time you would like a break or want to stop, let me know and we can do that.

**Topic 1 (Factors associated with development/identification/resolution of prescribing cascades); repeat questions 2 and 3 below for each prescribing cascade identified in the screening process**

1. The term ‘prescribing cascade’ is used to describe a situation when a drug is used to treat or prevent the side effect of another drug. What comes to mind when you hear the term *prescribing cascade*?
   1. What do you think of this type of situation? (e.g. appropriate? common?)
2. For you, we’re specifically interested in what led to you taking medication x (insert name of medication 1) and y (insert name of medication 2)
   1. Thinking back, when did medication x (use name) start? (e.g. year, timeframe)
   2. Tell me about who prescribed/started it, and why? (e.g. original prescriber, indication, symptoms being treated)
      1. How long was it intended for?
   3. How did you track whether x was working for you?
      1. How did you track if it was possibly causing any side effects? (or how did the prescriber, or someone else do this?)
   4. With regard to medication y (use name), tell me about when that was started?
   5. Who prescribed it and why?
   6. Sometimes, y is added to x to treat or prevent a side effect from x. In your case, it’s possible, though we don’t know for sure, that x was causing or had the potential to cause (name of symptom) that led to the prescribing of y. Do you think that could have been the case?
      1. Tell me why (or why not) you think that. (e.g. knew what to monitor for in terms of side effects, someone gave them information, found information etc.)
   7. When did you start to have (name of symptom for which y being used)?
      1. What did you do about it? (e.g. how symptom presented, what actions taken)
   8. What did the person who prescribed y at that time say about their impression of the situation? (e.g. what did they tell you? possible causes discussed? options for managing the symptom discussed?)
   9. How did the new medication y help – or not help – with the symptom?
   10. How did you keep track of whether it helped with the symptom? (e.g. see the prescriber again, check with someone else)
   11. With respect to the combination of x and y, has it ever been suggested that they might indicate a prescribing cascade?
       1. [If yes] how did that come to light? (e.g. who identified it? when? how?)
   12. How did that make you feel? (or How does it make you feel to think you might have a prescribing cascade?)
   13. About how long have you taken both x and y together?
       1. Are you still taking x and y?
   14. Have there been any dosage changes with either of them?
   15. What do you think needs to happen next with these two medications?

**Topic 2 (Clinical impact of prescribing cascades)**

1. We’re trying to get an understanding of how prescribing cascades affect you and how we should monitor for them. In particular, when y was added to x, what kinds of changes did you notice in how you felt? (e.g. function, symptoms, balance, falls, confusion)
2. And, if x or y was stopped, what kinds of changes did you notice (e.g. function, symptoms, balance, falls, confusion)

**Topic 3 (Potential interventions to prevent/identify/resolve prescribing cascades)**

1. Thinking back, what things might have helped uncover that one drug (y) was being used to treat (or prevent) the side effect of another drug (x)? (e.g. things the patient, a caregiver, the prescriber, another doctor, a pharmacist etc. could have done – like asking if a new symptom could have been caused by a drug, or giving a list of side effects to watch for, or doing a medication review)
2. What things do you think people need to do differently to prevent, manage or resolve prescribing cascades? (e.g. do something differently, learn something)
3. How might men and women differ in how they handle prescribing cascades or medications in general?
4. Would different kinds of strategies work more for men or women?
   1. Tell me more.

**Demographics:**

1. To help us put your responses in context with other cases, what is your age now?
   1. What was your age when this happened?
2. Tell me about your educational background. (e.g. highest level, type).
   1. How might that have impacted your awareness of your medications?
   2. How might that have impacted your awareness of the prescribing cascade?

Thank you for your time, and for your contribution to our research study.

We are looking for additional perspectives on the prescribing cascade that you experienced. If you have a family member who provides care for you who you feel would be suitable to provide another outlook on this prescribing cascade, would you like them to participate in this study?

[If yes, give patient a copy of Study Invitation Letter for Family Caregivers (Appendix G) to give to their family member]

[If no, continue to next prompt]

Would you like to provide us with contact information for a member of your healthcare team who you feel would be well-suited to provide us with more perspectives on the prescribing cascade you experienced? This person can be your family doctor, a specialist, your pharmacist, nurse, or other health care worker.

[If yes, record the health care provider’s contact information below after the patient has signed the “Consent to disclose personal health information document Oct 5 2018”]

[If no, thank the patient again and leave]

Health care provider’s full name, contact number or email: ____________________________________

**Prescribing Cascades Semi-structured Family Caregiver Interview Guide**

**Preamble**

Thank you for agreeing to participate in this study. My name is <insert name> and I am a research coordinator with the Bruyère Research Institute. Our study team is hoping to hear about <insert patient’s name>’s experiences with her/his medications, more specifically about the time when <insert patient’s name> was given a medication to manage a side effect from another of their medications. I am going to ask you a series of questions but want to assure you that there are no right or wrong answers. We are interested in your thoughts and experiences. The interview will last about 30 to 45 minutes but if at any time you would like a break or want to stop, let me know and we can do that.

**Topic 1 (Factors associated with development/identification/resolution of prescribing cascades); repeat questions 2 and 3 below for each prescribing cascade identified in the screening process**

1. The term ‘prescribing cascade’ is used to describe a situation when a drug is used to treat or prevent the side effect of another drug. What comes to mind when you hear the term *prescribing cascade*?
   1. What do you think of this type of situation. (e.g. appropriate? common?)
2. For (patient’s name), we’re specifically interested in what led him/her to taking medication x (insert name of medication 1) and y (insert name of medication 2)
   1. Thinking back, when did medication x (use name) start? (e.g. year, timeframe)
   2. Tell me about who prescribed/started it, and why? (e.g. original prescriber, indication, symptoms being treated).
      1. How long was it intended for?
   3. How did you or (patient’s name) track whether x was working?
      1. How did you track if it was possibly causing any side effects? (or how did the prescriber, or someone else do this?)
   4. With regard to medication y (use name), tell me about when that was started?
   5. Who prescribed it and why?
   6. Sometimes, y is added to x to treat or prevent a side effect from x. In your case, it’s possible, though we don’t know for sure, that x was causing or had the potential to cause (name of symptom) that led to the prescribing of y. Do you think that could have been the case?
      1. Tell me why (or why not) you think that. (e.g. knew what to monitor for in terms of side effects, someone gave them information, found information etc.)
   7. When did (patient’s name) start to have (name of symptom for which y being used)?
      1. What did he/she or you do about it? (e.g. how symptom presented, what actions taken)
   8. What did the person who prescribed y at that time say about their impression of the situation? (e.g. what did they tell you? possible causes discussed? options for managing the symptom discussed?)
   9. How did the new medication y help – or not help – with the symptom?
   10. How did you keep track of whether it helped with the symptom? (e.g. see the prescriber again, check with someone else)
   11. With respect to the combination of x and y, has it ever been suggested that they might indicate a prescribing cascade?
       1. [If yes] how did that come to light? (e.g. who identified it? when? how?)
   12. How did that make you feel? (or How does it make you feel to think (patient’s name) might have a prescribing cascade?)
   13. About how long has (patient’s name) taken both x and y together?
       1. Is he/she still taking x and y?
   14. Have there been any dosage changes with either of them?
   15. What do you think needs to happen next with these two medications?

**Topic 2 (Clinical impact of prescribing cascades)**

1. We’re trying to get an understanding of how prescribing cascades affect people and how we should monitor for them. In particular, when y was added to x, what kinds of changes did you notice in how (patient’s name) felt? (e.g. function, symptoms, balance, falls, confusion)
2. And, if x or y was stopped, what kinds of changes did you notice (e.g. function, symptoms, balance, falls, confusion)

**Topic 3 (Potential interventions to prevent/identify/resolve prescribing cascades)**

1. Thinking back, what things might have helped uncover that one drug (y) was being used to treat (or prevent) the side effect of another drug (x)? (e.g. things the patient, a caregiver, the prescriber, another doctor, a pharmacist etc. could have done – like asking if a new symptom could have been caused by a drug, or giving a list of side effects to watch for, or doing a medication review)
2. What things do you think people need to do differently to prevent, manage or resolve prescribing cascades? (e.g. do something differently, learn something)
3. How might men and women differ in how they handle prescribing cascades or medications in general?
4. Would different kinds of strategies work more for men or women?
   1. Tell me more.

Thank you for your time, and for your contribution to our research study. Your input is greatly appreciated!

**Prescribing Cascades Semi-structured Healthcare Provider Interview Guide**

**Notes for interviewers:** For the purposes of this guide, healthcare providers invited to participate are one of the following people who were identified by a patient as being able to speak about the patient’s experiences with prescribing cascades: family doctor, a specialist, pharmacist, nurse, or other health care worker. Herein, they will be called “healthcare providers”

We recognize that there seem to be more questions than are possible to answer within a 15-30 minute time period. We anticipate that most health care providers will not be able to recall when or how prescribing cascades evolved so some questions will result in very short answers. However, we’ve been inclusive of a variety of possible questions. If the interview reaches 30 minutes, the interviewer will explain that the allotted time is complete and ask permission to continue (anticipating that this may not be possible in some situations).

**Preamble**

Thank you for agreeing to participate in this study. My name is <insert name> and I am a research coordinator with the Bruyere Research Institute. Thank you for taking the time to speak today. You were invited to participate in this study because (patient name) identified you as being able to speak to their experience with one or more possible prescribing cascades. (Patient name) was identified as someone who may have had a prescribing cascade, a situation where a drug might be being used to treat the side effect of another drug. (Patient name) has already been interviewed and gave written permission for us to contact you to understand more about whether a prescribing cascade has happened, how it might have come about and how it might be resolved.

I have a series of questions; I’m not looking for particular answers and nothing will be viewed as correct or incorrect in your responses. The more you are willing to share will help our team as a longer-term goal is to build tools, strategies or interventions that can assist with identifying, preventing or managing prescribing cascades in the future. The interview will last about 20 to 30 minutes but if at any time you would like a break or want to stop and continue at another time, let me know and we will make arrangements.

**Topic 1 (Factors associated with development/identification/resolution of prescribing cascades); repeat questions 2 and 3 below for each prescribing cascade identified in the screening process (as time allows)**

1. To get started, tell me about what you have seen with regard to prescribing cascades in general? (e.g. types)
2. For (patient name), we’re specifically interested in what led to he/she being prescribed medication x (insert name of medication 1) and y (insert name of medication 2)
   1. Thinking back, when was medication x (use name) first started? (e.g. year, timeframe)
   2. Tell me about who prescribed/started it, and why? (e.g. original prescriber, indication, symptoms being treated);.
      1. How long was it intended for?
   3. How did you (or the original prescriber) track whether x was working?
      1. How did you track if it was possibly causing any side effects? (or how did the prescriber, or someone else do this?)
   4. With regard to medication y (use name), tell me about when that was started.
      1. Who was the original prescriber?
      2. What were the circumstances surrounding medication y being prescribed? (some type of symptom presented itself?)
   5. Sometimes, y is added to x to treat or prevent a side effect from x. In this case, it’s possible, though we don’t know for sure, that x was causing or had the potential to cause (name of symptom) that led to the prescribing of y. Do you think that could have been the case?
      1. Tell me why (or why not) you think that. (e.g. knew what to monitor for in terms of side effects, someone gave them information, found information etc.)
   6. When did (patient’s name) start to have (name of symptom for which y being used)?
      1. What did you (or the patient, or another prescriber) do about it? (e.g. how symptom presented, what actions taken)
   7. What did the person who prescribed y at that time say about their impression of the situation? (e.g. what did they tell you? possible causes discussed? options for managing the symptom discussed?)
   8. How did the new medication y help – or not help – with the symptom?
   9. How did you keep track of whether it helped with the symptom? (e.g. asked the patient to call, or come for an appointment)
   10. With respect to the combination of x and y, has it ever been suggested that they might indicate a prescribing cascade?
       1. [If yes] how did that come to light? (e.g. who identified it? when? how?)
   11. How did that make you feel? (or How does it make you feel to think the patient might have a prescribing cascade?)
   12. About how long has (patient’s name) taken both x and y together?
       1. Is he/she still taking x and y?
   13. Have there been any dosage changes with either of them?
   14. What do you think needs to happen next with these two medications?

**Topic 2 (Clinical impact of prescribing cascades)**

1. We’re trying to get an understanding of how prescribing cascades affect patients and how we should monitor for them. In particular, when y was added to x, what kinds of changes did you notice in how the patient felt? (e.g. function, symptoms, balance, falls, confusion)
2. And, if x or y was stopped, what kinds of changes did you notice (e.g. function, symptoms, balance, falls, confusion)

**Topic 3 (Potential interventions to prevent/identify/resolve prescribing cascades)**

1. Thinking back, what things might have helped uncover that one drug (y) was being used to treat (or prevent) the side effect of another drug (x)? (e.g. things the patient, a caregiver, the prescriber, another doctor, a pharmacist etc. could have done – like asking if a new symptom could have been caused by a drug, or giving a list of side effects to watch for, or doing a medication review)
2. At the individual patient-provider level, practice level and health care system level, what do you think needs to change to increase awareness about prescribing cascades with the goal of preventing, managing or resolving them?
3. How might men and women differ in how they handle prescribing cascades or medications in general?
4. Would different kinds of strategies work more for men or women?
   1. Tell me more.

Thanks very much for your time. It’s been much appreciated. We’ll be collating results from all of our interviews and would be happy to let you know when the final overall results are available.
