# Supplementary material for: “Kind of blurry”: Deciphering clues to prevent, investigate and manage prescribing cascades
Source: PLoS One. 2022 Aug 31;17(8):e0272418. doi: 10.1371/journal.pone.0272418 (PMC9432713; doi:10.1371/journal.pone.0272418)
Supplement: S1 File — (DOCX) [file pone.0272418.s004.docx]

**“Kind of blurry”: Deciphering clues to prevent, investigate and manage prescribing cascades**

**ONLINE SUPPLEMENTARY MATERIAL**

**SUPPLEMENTARY FILE S4: Exported Code Book**

Authors: Barbara Farrell, Emily Galley, Lianne Jeffs, Pam Howell, Lisa M. McCarthy

Contact: lisa.mccarthy@utoronto.ca

Prescribing Cascades (Full)

Nodes\\Revised (November 2019; exported January 2020)

| Name | Description |
| --- | --- |
| **Caregivers-Family** | **Currently a minimally-defined 'bin' to capture any data relating to caregivers and/or family members, both regarding a specific case and more generally.** |
| Involvement | - Participant's experiences with, attitudes towards, evaluations of, etc family member and/or caregiver involvement with medication management |
| Knowledge | - Participant’s experiences with, attitudes towards, evaluations of, etc family caregiver knowledge regarding medication management |
| **Cascades** | **Contains all codes directly related to prescribing cascades including knowledge, experiences, attitudes, and activities.** |
| Appropriate (HCP) | - HCP (health care professionals) experiences of, attitudes towards, potentially appropriate prescribing cascades both generally and in particular cases - Patient/resident equivalent falls under 'Positive views of RxC' |
| Familiarity RxC | - Participant familiarity, or unfamiliarity, with the terminology, concept, etc of cascades - Does not refer to knowledge of case-specific suspected/ identified cascades |
| HCP collaboration & communication | - Instances/examples of collaboration between HCPs - Descriptions of collaborative/communication processes or protocols between HCPs |
| Impact of RxC | - Codes for patient or HCP stating whether or not the prescribing cascade had any impact on them - Patient or HCP descriptions of HOW a cascade has had an impact/WHAT the impact is/suspected to be |
| Inappropriate (HCP) | - HCP experiences of, attitudes towards, potentially inappropriate prescribing cascades both generally and in particular cases - Approximate patient equivalent is "Negative views of RxC" |
| ***Views of RxCascades*** *(Positive and Negative views of RxC [residents/patients]) are collapsed into this code)* | *- Participants’ negative attitudes towards concept of prescribing cascades - Participants’ negative views towards their own suspected/identified prescribing cascade*  *- Participants’ positive attitudes towards concept of prescribing cascades - Participants’ positive views towards their own suspected/identified prescribing cascade* |
| Prevention | - Experiences, attitudes, considerations, suggestions addressing how prescribing cascades may be prevented - Applies to both patients/residents and HCPs |
| Process of Identification | - Captures steps involved in identifying and confirming the presence of a prescribing cascade in a given case |
| Deciphering medications (HCP) | - HCP process/experience of figuring out when, why, etc a given medication was started for a given patient in the context of a suspected/confirmed prescribing cascade; roughly stands in for medication history - Distinguished from ‘Uncertainty – starting med’ in its reference to action, i.e., deciphering refers to the activity of determining information about a given medication |
| Identification of RxC | - Process of identifying/confirming a suspected prescribing cascade - General challenges described in identifying/confirming prescribing cascades both in general and in a given case |
| Identifier of RxC | - Person(s) who identified a suspected/confirmed prescribing cascades in a given case |
| Uncertainty - continuing need for meds (HCP) | - HCP experiences regarding assessing the continued need for a given medication, particularly where the medication was prescribed by someone other than themselves |
| Uncertainty - starting meds (HCP) | - HCP uncertainty regarding when and why a given medication was started for a given patient/ resident in the context of a suspected/confirmed prescribing cascade - Refers to lack of certainty regarding the information available about a given medication, its purpose, etc |
| Use of health records  *(Use of EMRs has been collapsed into this code)* | - Experiences of, attitudes towards, the role of electronic medical records in identifying and resolving prescribing cascades  *- Experiences of, attitudes towards, the role of health records in identifying and resolving prescribing cascades* |
| Process of resolving | - Descriptions of how an identified cascades has been/may be resolved - Challenges described in resolving an identified cascade or in resolving cascades in general |
| Outcomes | - Results of attempts to resolve cascades; includes both successful and unsuccessful attempts |
| Risk-benefit analysis (HCP) | - HCP experiences of weighing potential risks and benefits of maintaining a suspected/ identified prescription cascade for a given patient |
| Suspected RxC | - Patient or HCP thoughts about whether a prescribing cascade exists, details a suspected cascade |
| Willingness to manage RxC (RP) | - Patient’s willingness to maintain a suspected/identified prescribing cascade in the context of managing their symptoms, achieving health priorities - For example, a patient may be experiencing a cascade but does not have an issue with maintaining the status quo because their symptoms are being managed to their satisfaction |
| **Gender** | **- Currently undifferentiated bin to hold any fragments pertaining to gender** |
| **HCP Knowledge** | **Contains all codes relating to HCP knowledge of medications, both in regards to a specific case/cascade being discussed as well as more general medication literacy.** |
| Educational impact (HCP)  *(Drug metabolism (HCP) is collapsed into this code)* | - Impact perceived by HCPs of education and/or training on understanding/ knowledge/awareness of prescribing cascades and relevant medication literacy - For example, a HCP might relate that a physician specifically trained in geriatrics would have a greater capacity to identify problematic medications in an older patient  *- Knowledge/awareness on the part of HCPs regarding changes to drug metabolism resulting from resident/patient aging* |
| Medication side effects (HCP) | - HCP knowledge and/or awareness of potential side effects of a given medication |
| **Medication Management** | **Contains codes covering aspects of medication management from both residents/patients and HCP participants. Distinguished from Medication Knowledge by active nature of codes: they describe feelings, impacts, activities/actions, and other attitudes, circumstances, etc which influence how participants approach and engage with medications, both those specific to the case being discussed and with medications/the use of medications in general** |
| Alternatives | - Use of alternative treatment options (i.e., non-pharmaceutical) in addressing symptoms and/or side-effects in the context of a suspected/identified cascade - For example, opting for a ginger supplement instead of Gravol or cannabis rather than opioids |
| Belief in benefits (RP) | - Patient’s expressions of belief that they experience benefits from taking a particular medication - e.g. “I’m not sure what medications do but I feel better when I take them so they must be doing something good” - this may be treated as the ‘subjective’ partner of ‘Drug effectiveness’ |
| **Changing meds - negative feelings** *(Changing meds – negative feeling (HCP) and (RP) are collapsed into this code)* | *- Hesitancy, reluctance, anxiety, fear on the part of the HCP to make changes to medications that a patient is currently taking*  *- Hesitancy, reluctance, anxiety, fear on the part of the patient to make changes to the medications they are current taking* |
| Deprescribing | - Knowledge and experiences of deprescribing medications particularly in the context of a suspected/identified cascade |
| Drug effectiveness | - Experiences of effectiveness (i.e., in addressing target symptoms) of particular drugs in context of suspected/identified prescribing cascade - e.g. “I know Drug B is working because I haven’t had the symptom Drug B is meant to address since I started taking it” - As well, evidence of effectiveness - this may be treated as the ‘objective’ partner of ‘Belief in benefits’ |
| Feelings towards meds (RP) | - Patient’s feelings towards their own medications as well as towards the idea of taking medications in general - May be positive, negative or neutral - Distinguished from ‘Patient Preferences’ in focus on feelings; for example, a patient may prefer not to be on so many medications but feel positively about their own medications for a given reason |
| GOALS OF CARE | - Describes patient and HCP goals in medication management, treatment choices, etc - Distinguished from 'Impact of patient priorities' in that priorities relate to how goals are determined and/or achieved; e.g. a patient may prioritize not having disruptive side effects over potential longevity and the goal becomes to maximize patient comfort |
| Impact of past experiences | - Instances where participant’s past experiences with medication, healthcare providers, medical treatment, etc have, or are suggested to have, an impact on residents/patients and/or HCP willingness to make changes to medications |
| Impact of patient priorities on HCP decisions | - Impact of resident/patient preferences regarding their medications and treatment plans on HCP decision making, particularly with regards to making changes to medications |
| Initiative and Control | - Incidents of patient exerting control or taking initiative over their treatment particularly with regards to their medications - May also code for attitudes towards exerting control or taking initiative - For example, a patient who seeks treatment alternatives, proactively addresses their symptoms (e.g. adding supplements, monitoring blood sugar) |
| Non-adherence | - Describes instances in which a patient is/did not take their medications as indicated by an HCP - For example, a patient relating that they did not want to take a medication anymore and so stopped doing so without discussion with an HCP |
| Patient preferences | - Patient’s preferences regarding taking specific medications, i.e., their current or past medications - Patient’s preference regarding taking medications in general; e.g., their preference for not taking medications at all or for seeking/using non-pharmaceutical treatment options, their preference for certain types of medication/treatment over others (e.g. taking insulin instead of medications, taking pills instead of needles) - distinguished from ‘Feelings towards meds’ |
| Temporal challenges | - Challenges described in relating a given emergent symptom/side effect/sign to specific medication(s) due to the time elapsed between starting the medication(s) and emergence of symptom/side effect/sign - May also code for descriptions of the general ease or difficulty experienced in making these links where a significant amount of time, or what the subject views as a significant amount to time, has passed between starting a given medication and the emergence of symptoms/side effects/signs |
| Understanding on-going benefit | - Challenges describe in determining whether a given medication remains beneficial in addressing a target symptom/condition/etc - Challenges described in understanding on-going benefit, or lack thereof, of a current medication - for example, a patient has been taking a given medication for many years and it is no longer clear what, if any, benefit they are deriving from it. This patient may have trouble understanding or accepting that they may no longer need such a medication |
| **Patient Knowledge** | **Contains all codes relating to resident/patient knowledge regarding their medications as well as their general medication literacy. Distinguished from Medication Management in being 'fact-based' i.e., unrelated to feelings, attitudes, or experience. The one exception to this is 'Educational Impact' which is included due to its relationship to medication literacy. - INCLUDES HCP knowledge, perceptions, etc regarding patient knowledge** |
| Educational impact (RP) | - Impact perceived by patients of their level of education and their medication literacy/capacity to understand and discuss their medications |
| General knowledge (RP) | - Patient's general knowledge regarding their own medications or medication regime - Does not (currently) refer to broader medication literacy; focused on situation-specific knowledge |
| Knowledge of OTC meds | - Patient knowledge regarding OTC meds including their potential side-/effects, status as drugs, etc |
| Medication duration (RP) | - Patient knowledge regarding how long they have been taking specific medications - Patient knowledge of how long a given medication is meant to be taken |
| Medication prescriber (RP) | - Patient knowledge regarding who first prescribed specific medications currently being taken |
| Medication side effects (RP) | - Patient knowledge and/or awareness of potential side effects of a given medication they are currently taking or took in the past - For example, information they describe having received from a prescriber or other HCP about potential side effects of a given medication |
| Starting meds (RP) | - Patient knowledge regarding when specific medications/treatment plans were started and why they were started |
| **Patient-HCP Relationship** | **Contains all codes describing relationships between patients/residents and HCPs, particularly with regards to medication management and prescribing cascades. Some codes may be specific to a given relationships (i.e., part of the case being discussed) while others might be more generally applied.** |
| Asking questions | - Incidents related by patient of asking questions about their medications, medication management, symptoms/side effects, treatment, etc - Incidents of HCPs being asked questions by their patients regarding latter’s medications, medication management, symptoms/side effects, treatment, etc - codes patient’s attitudes (positive) towards asking questions of HCPs |
| Following instructions | - Patient attitudes and experiences regarding following HCP instructions regarding medications - May pertain to the patient’s own experiences or more generally to the idea of following HCP instructions |
| Not asking questions | - Incidents related by patient of refraining from asking questions about their medications, medication management, symptoms/side effects, treatment, etc - Patient attitudes (negative or neutral) towards asking questions about their medications |
| Patient-HCP communication | - Patient experiences and attitudes regarding communicating with HCPs in the context of information about their medications, medication management, symptoms/side effects, treatment, etc - Also the reverse, i.e., HCP experiences and attitudes regarding communication with patients/residents about their medications, medication management, symptoms/side effects, treatment, etc - Includes lack of communication, communication issues (e.g. misunderstandings) successful communication with HCPs |
| Trust in HCPs | - Patient feelings and experiences of trust towards healthcare providers - Patient attitudes towards HCPs’ decisions regarding their medications, medication management, symptoms/side effects, treatment, etc |
| **Side Effects and Symptoms** | **Contains codes pertaining to the reporting, identification, and treatment of side effects, symptoms, and signs. Captures EXPERIENCES of side effects, symptoms, and signs reported by both residents/patients and HCPs. Does not code for KNOWLEDGE of side effects** |
| Monitoring and Identification SES | - Identification of side effects by either patients/residents or HCPs - Challenges experienced, particularly by HCPs, in distinguishing between side effects and new symptoms, e.g. determining whether nausea is a side effect of a current medication or a symptom of a new/changing condition - Monitoring symptoms/side effects for goal of identification/treatment |
| Reported SES | - Experiences of side effects described or related by patient, particularly with reference to a specific medication; note that they do not have to be considered/perceived as side effects by the patient - Also codes for patient reporting no side effects, i.e., responding to questions about side effects in the negative - Side effects described or related by HCP in reference to the experiences of a specific patient with a specific medication - As above, also codes for the reported absence of side effects |
| Treatment and Investigation SES | - Experiences and challenges of assessing, monitoring, treating side effects - May also code for the investigation of symptoms/side effects/signs i.e., to determine their cause, as well as the reverse (choosing treatment over investigation) or both (simultaneous treatment and investigation) |
