# Supplementary material for: “Kind of blurry”: Deciphering clues to prevent, investigate and manage prescribing cascades
Source: PLoS One. 2022 Aug 31;17(8):e0272418. doi: 10.1371/journal.pone.0272418 (PMC9432713; doi:10.1371/journal.pone.0272418)
Supplement: S2 File — (DOCX) [file pone.0272418.s005.docx]

**“Kind of blurry”: Deciphering clues to prevent, investigate and manage prescribing cascades**

**ONLINE SUPPLEMENTARY MATERIAL**

**SUPPLEMENTARY FILE S5: Coding Summaries**

Authors: Barbara Farrell, Emily Galley, Lianne Jeffs, Pam Howell, Lisa M. McCarthy

Contact: lisa.mccarthy@utoronto.ca

**Legend:** CG (caregiver), GDH (Geriatric Day Hospital), HCP (healthcare provider), OTC (over-the-counter), RxCascades (prescribing cascades)

| **Code Name & Description** | **Summary** |
| --- | --- |
| ***Caregivers and Family*** *(includes* Knowledge *and* Involvement*)*  Currently a minimally-defined 'bin' to capture any data relating to caregivers and/or family members, both regarding a specific case and more generally. Participant's experiences with, attitudes towards, evaluations of, etc family member and/or caregiver knowledge and involvement with medication management | Caregivers and family members had a widely varying degree of involvement in and knowledge of patient medication experiences and management. In some cases, patients and caregivers indicated a high degree of involvement and HCPs indicated the importance of involving caregivers. However, some patients described having no family members involved in their care due to a variety of reasons including estrangement and incapacity. Involvement in care did not necessarily correlate with greater knowledge regarding a patients’ medications even where caregivers had healthcare experiences themselves, and caregivers expressed an interest in, and need for, more and better information about their patient/family members’ specific cases. |
| ***HCP Knowledge***  Contains all codes relating to HCP knowledge of medications, both in regards to a specific case/cascade being discussed as well as more general medication literacy. | Education and environment were identified as factors influencing HCP knowledge about polypharmacy, medication management, and prescribing cascades. |
| Educational Impact: Impact perceived by HCPs of education and/or training on understanding/ knowledge/awareness of prescribing cascades and relevant medication literacy - For example, a HCP might relate that a physician specifically trained in geriatrics would have a greater capacity to identify problematic medications in an older patient | Respondents identified ways in which formal education influenced the contrasting approaches of physicians and pharmacists to medication issues, including the recognition, investigation, and management of prescribing cascades: while physician education was described as encouraging a disease-focused approach to medication management, often resulting in a failure to identify medication side-effects and the consequent prescription of addition drugs, pharmacists’ more in-depth knowledge of pharmaceuticals and their effects was perceived as being conducive to a drug-focused approach in which emergent symptoms were more likely to be identified as potential side effects of medication rather than as indications of a new medical condition. |
| Side effects*:* HCP knowledge and/or awareness of potential side effects of a given medication | Physicians in particular appeared to lack sufficient knowledge and understanding of medication side effects, resulting in a reduced capacity to identify emergent signs and symptoms as potential side effects and to connect them to causative drugs. This inability to identify side effects was connected to incapacity to recognize potential prescribing cascades and with treating emergent signs and symptoms as indicators of new conditions, contributing to increased drug use rather than investigation. |
| General (no sub-code) | Respondents who worked in geriatric and/or multidisciplinary practices described their environment as influential on their approach to, and knowledge of, medication management, suggesting that practical experience and interprofessional collaboration may also be important in determining HCP ability/capacity to recognize, investigate, and manage problematic polypharmacy and prescribing cascades. |
| ***Patient Knowledge***  Contains all codes relating to resident/patient knowledge regarding their medications as well as their general medication literacy. Distinguished from Medication Management in being 'fact-based' i.e., unrelated to feelings, attitudes, or experience. The one exception to this is 'Educational Impact' which is included due to its relationship to medication literacy. INCLUDES HCP knowledge, perceptions, etc regarding patient knowledge | Patients demonstrated an inconsistent level of knowledge and understanding regarding their medications; gaps or absences in knowledge included reasons for use (initiation and continuation), history of use, names of medications, and original prescribers. Some patients demonstrated minimal medication literacy including the general potential for side effects of medications and the risks of polypharmacy. With regards to their own medications, many patients were able to broadly describe their purpose but demonstrated a lack of more in-depth knowledge or understanding. |
| Education: Impact perceived by patients of their level of education and their medication literacy/capacity to understand and discuss their medications | Some patients with higher reported levels of educational attainment also reported significant, independent efforts to seek and obtain information about their medications, i.e., they did not rely exclusively on their HCP to provide them with information. However, given the small ‘sample’ size, no conclusion can really be drawn. |
| OTC medications: Patient knowledge regarding OTC meds including their potential side-/effects, status as drugs, etc | HCPs described generally poor knowledge on the part of patients regarding over-the-counter medications and their potential side effects, interactions, and status as drugs. This last point was cited as a barrier to HCP knowledge of patient medication experiences, i.e., since patients might not consider OTC medications to be drugs, they would not report their use to HCPs. Very little data specific to OTC though. |
| Starting meds and Medication duration: Patient knowledge regarding when specific medications/treatment plans were started and why they were started. Patient knowledge regarding how long they have been taking specific medications. Patient knowledge of how long a given medication is meant to be taken | Patients could typically, though not uniformly, recall at least approximate time-lines and reasons for their medications, i.e., about how long they’d been taking a specific medication, points in time at which changes in dose of a specific medication had occurred. Few seemed to know, or have been informed, how long they could expect to be on a specific medication at the time it was prescribed. |
| Side effects*:* Patient knowledge and/or awareness of potential side effects of a given medication they are currently taking or took in the past - For example, information they describe having received from a prescriber or other HCP about potential side effects of a given medication | The largest knowledge gap was around potential side effects and problematic polypharmacy: patients often described little to no knowledge of the possible side effects of their medications and reported that they had never received information about them from their HCPs or that they could not recall receiving this information. Patients who related the highest amount of knowledge and understanding of their medications described significant personal efforts to find information, conducting independent research, seeking alternatives to perceived issues, and actively engaging their HCPs in discussion. Other patients described being heavily, or exclusively, reliant on their HCPs to provide the necessary information and felt that it was incumbent on HCPs to do so. |
| General  *Coding description*: Patient's general knowledge regarding their own medications or medication regime. Does not (currently) refer to broader medication literacy; focused on situation-specific knowledge | Patients who demonstrated lower degrees of knowledge and/or understanding of their medications largely expressed an interest in knowing more and frustration at their current degree of knowledge. Amongst the challenges they described in increasing their knowledge were lack of communication from HCPs on the details of medications; lack of easily accessible and understandable information; trouble recalling information provided; being in a position of being both patient and caregiver to another patient; and the use of blister packs which were also associated with a loss of information. Some patients related their lack of knowledge to the high degree of trust which they bestowed on their HCPs, i.e., patients didn’t have to know about their medications because they trusted their physicians in particular to make the right decisions for them. HCP also commented about what patients don’t know about their medications and what they felt they should know. |
| ***Patient-HCP Relationships***  Contains all codes describing relationships between patients/residents and HCPs, particularly with regards to medication management and prescribing cascades. Some codes may be specific to a given relationships (i.e., part of the case being discussed) while others might be more generally applied. |  |
| Trust (includes Asking/Not asking questions and Following instructions): Patient feelings and experiences of trust towards healthcare providers. Patient attitudes towards HCPs’ decisions regarding their medications, medication management, symptoms/side effects, treatment, etc. Incidents related by patient of refraining from/asking questions about their medications, medication management, symptoms/side effects, treatment, etc. Patient attitudes towards asking questions about their medications. Patient attitudes and experiences regarding following HCP instructions regarding medications. May pertain to the patient’s own experiences or more generally to the idea of following HCP instructions | Trust in HCPs on the part of patients was often framed in the context of knowledge: patients who described a high degree of trust in HCPs also described the latter as expert while relationships of distrust were described in parallel to doubts regarding HCP expertise. Trust in physicians in particular was connected, in some cases, with the perceived effectiveness of care received; for some patients, prolonged, negative experiences of care were related (by the patients themselves) to a lack or loss of trust in their physicians.  Trust relationships also manifested differently depending on whether the patient was dealing with a physician or a pharmacist. In relationships with physicians, trust was demonstrated by not asking questions with pharmacists, patients described trusting relationships as being those in which they felt comfortable asking questions. Pharmacists were described by many respondents (patients, HCPs, and CGs) as being particularly valuable sources of information and expertise when it came to medication management, polypharmacy, and prescribing cascades. |
| Patient-HCP Communication: Patient experiences and attitudes regarding communicating with HCPs in the context of information about their medications, medication management, symptoms/side effects, treatment, etc. Also the reverse, i.e., HCP experiences and attitudes regarding communication with patients/residents about their medications, medication management, symptoms/side effects, treatment, etc . Includes lack of communication, communication issues (e.g., misunderstandings) successful communication with HCPs. | Patients who reported positive relationships with their HCPs did not appear to be better informed regarding their medications than those who reported negative experiences, suggesting that positively-viewed relationship did not necessarily result in better communication. In at least one case, negative relationship experiences resulted in a high degree of personal initiative on the part of the patient to seek and obtain their own information about their medications rather than relying on their HCPs. Patients who participated in the GDH program reported a higher quantity and quality of communication with HCPs within the program than they were accustomed to, suggesting an inferior standard of communication in their non-GDH HCP experiences. |
|  | NOTE  An interesting note on data: in most cases, we were not able to speak with the original prescriber of certain drugs, particularly where the drug had been part of the patient’s regime for many years or decades. So where patients reported receiving little or no information about their drugs when they were prescribed, we do not know the physician’s experience or perception of the initial prescription. This would have been particularly interesting in cases where patients could not recall whether they had been provided with information. |
| ***Symptoms and Side-effects*** |  |
| Treatment & Investigation: Experiences and challenges of assessing, monitoring, treating side effects . May also code for the investigation of symptoms/side effects/signs i.e., to determine their cause, as well as the reverse (choosing treatment over investigation) or both (simultaneous treatment and investigation). | Investigation of side effects and, by extension, possible prescribing cascades, often appeared to be inhibited by a failure to consider emergent signs and symptoms as possible side effects of one or more medications. In some cases, this resulted in multi-year investigations involving numerous physicians and diagnostic tests. In others, it was the patient themselves who made the connections between the symptoms being experienced and their current medications. Where symptoms were suspected to be side effects, HCPs described difficulty in connecting them to specific medications particularly where a patient was experiencing significant polypharmacy (i.e., side effect could be caused by multiple, or a combination, of medications) or where the side effect was particularly common (e.g., nausea.) Investigation of symptoms and side effects, and consideration of the possible experience of a prescribing cascades, is likely (?) related to physician knowledge. |
| Monitoring: Identification of side effects by either patients/residents or HCPs. Challenges experienced, particularly by HCPs, in distinguishing between side effects and new symptoms, e.g., determining whether nausea is a side effect of a current medication or a symptom of a new/changing condition. Monitoring symptoms/side effects for goal of identification/treatment. | Monitoring of medications appeared to focus primarily on drug effectiveness rather than on safety or side effects, i.e., patients would be asked about the symptom the drug was intended to address but not about potential side effects. The intensity of monitoring was also reported to be related to the amount of time a patient had been taking a given drug: more recently prescribed medications were more closely monitored than long-term prescriptions. Monitoring also depended on patients reporting emergent symptoms to HCPs and where there were no patient complaints, HCPs were less likely to investigate the possibility of side effects. There was a lack of awareness about what side effects to monitor for, how to think about a symptom as a side effect when the symptom is vague or when multiple drugs might be contributing. |
| ***Cascades***  Contains all codes directly related to prescribing cascades including knowledge, experiences, attitudes, and activities. |  |
| Familiarity with RxCascades: participant familiarity, or unfamiliarity, with the terminology, concept etc of cascades – does not refer to knowledge of case-specific/identified cascades | There is little awareness amongst patients/caregivers of the term “prescribing cascades” though the concept of medications causing side effects treated by other medications is not unknown. After the concept was explained, people seemed to understand it though it was unclear if they could apply that understanding for themselves. Amongst physicians, awareness of the concept/term/examples is slowly increasing though there does not seem to be a common understanding or approach to identifying/resolving them. Awareness amongst physicians seems to be influenced by working with certain people (e.g GDH pharmacist) and also by being involved in the study. |
| Suspected RxCascades: patient or HCP thoughts about whether a prescribing cascade exists, details a specific cascade | [Not reviewed in detail; suggest we develop a chart (like for the pilot) with a row for each patient, indicating the suspected prescribing cascade(s) – ie. reason for study referral, the pharmacist impression of the cascade(s) and the patient and caregiver’s impression of the cascade(s), including how certain people were about identification and steps toward resolution (including if resolved or not).] |
| Process of identification: deciphering medications (HCP): HCP process/experience of figuring out when, why, etc a given medication was started for a given patient in the context of a suspected/confirmed prescribing cascade; roughly stands in for medication history – distinguished from ‘uncertainty – starting med’ in its reference to action, i.e., deciphering refers to the activity of determining information about a given medication. | The process of investigating whether a prescribing cascade has occurred involves a number of steps largely concerning identifying the sequence of events (the timing of medications and appearance of signs/symptoms) and the purpose of medications. Challenges include not knowing the reasons for the prescribing of a medication, the name of the original prescriber, how long the patient has been taking the medication and which medications were started in what order. Also unclear is whether a medication might be worsening a pre-existing condition already being treated by a drug. Also unclear is whether a sign or symptom might be contributed to by a number of medications, and how to sort out which of those medications are most likely contributing. These challenges contribute to the uncertainty around whether a prescribing cascade has truly occurred and which medications might be implicated as causative agents.  One physician commented that their involvement in the study made them more alert to the possibility of a prescribing cascade and that this affected their prescribing cascade identification thought process.  Physicians and pharmacists approach the investigation of prescribing cascades to varying depths with divergent assumptions. |
| Process of identification: identifier of cascade: person(s) who identified a suspected/confirmed prescribing cascade in a given case | There is very little coded data related to the ‘identifier’ of cascades. Patients, pharmacists, family doctors, naturopaths and specialists were involved in identifying the possibility of a prescribing cascade. Sometimes, multiple people were involved (including, for example, patient s conferring with their pharmacist or family doctor, and several specialists being involved in investigating the issue before a medication was identified as a potential cause). Comments are made about people not taking the responsibility to resolve a cascade when it’s thought possible “no one is kind of taking responsibility to actually resolve problems that are hinted at, in this case with the gastroenterologist saying stop your metformin. And people might argue, well, it’s not the gastroenterologist’s place to stop a diabetes medication. But if he’s investigating the side effect of the medication, then maybe it is the specialist’s place to do that.” |
| Process of identification: identification of cascade: Process of identifying/confirming a suspected prescribing cascade - General challenges described in identifying/confirming prescribing cascades both in general and in a given case | As in the ‘deciphering’ section above, the process of trying to identify prescribing cascades involves investigative work. People speak about ‘teasing out information’, finding ‘clues’ and ‘deciphering’. Challenges are many and include limited or lost history (about reasons for medications, duration, original prescriber), cognitive impairment, medications having multiple possible reasons for use and original indication being unclear, and signs or symptoms that may be impacted by multiple medications a patient is taking. Limited time for investigation and detective work, as well as a medical mindset (professional culture approach, heuristic) that leans toward making a provisional diagnosis and treatment can result in not identifying medications as possible contributors to a problem or a superficial identification of the most likely drug to be contributing versus a combination of drugs that might be contributing to a problem. Patients speak about seeing multiple specialists who do not necessarily link symptoms to medications as potential causes but carry out many tests and investigations. Poor communication amongst health care providers and a reluctance to change medications impacts on the ability to identify prescribing cascades.  These challenges create (layers of) **uncertainty** about confirming the existence of prescribing cascades. Interviewees talk about how prescribing cascades do not necessarily follow a ‘classic’ linear approach. Vague symptoms can sometimes be caused by multiple drugs; not being able to stop each of those drugs makes it difficult to say a prescribing cascade has happened with certainty. Several medications can cause a constellation of symptoms which might be treated by different types of other medications. Varying knowledge and approaches amongst health care providers results in differing opinions about prescribing cascades and subsequent management.  Knowledge about how drugs work and how dose responses may change over time (ie. with age) facilitates identification of medication causes. Interprofessional discussions are also helpful to share perspectives |
| Process of identification: uncertainty – starting meds (HCP): HCP uncertainty regarding when and why a given medication was started for a given patient/resident in the context of a suspected/confirmed prescribing cascade – refers to lack of certainty regarding the information available about a given medications, its purpose etc | Uncertainty about the original reason for a medication (and difficulty finding this information) hampers the ability to assess the effectiveness of a medication. This makes it difficult to conduct a risk/benefit analysis of continuing or stopping a medication and thus the identification and management of a possible prescribing cascade. |
| Process of identification: uncertainty – continuing need for meds (HCP): HCP experiences regarding assessing the continued need for a given medication, particularly where the medication was prescribed by someone other than themselves | Even when the original indication for a medication is relatively clear (e.g., furosemide for ‘fluid on the lungs’), reasons for its’ ongoing use may be less clear. |
| Process of identification: use of health records: experiences of, attitudes towards, the role of electronic health records in identifying and resolving prescribing cascades | Including information about reasons for medication use, the duration of medication use and a history of medication intolerances in electronic health records would facilitate more efficient information retrieval and decision-making. Some electronic records have the capacity to document some of these information components but they are not necessarily used to their full capacity, may be ‘clunky’ to use and do not contain historical information prior to the implementation of electronic records. Going back to paper charts for information is most often not feasible. |
| HCP collaboration and communication: instances/examples of collaboration between HCPs – descriptions of collaborative/communication processes or protocols between HCPs | Interviewees described efforts to communicate with health care provider colleagues by fax, on paper, by phone and by providing questions for patients to ask of their health care providers. The sharing of current medication lists, reasons for medication prescribing or changes (sometimes written directly on the prescriptions), questions about medication use/benefit and plans for next medication changes amongst and between patients, family doctors, specialists, community pharmacists and hospitalists are considered valuable for patient care; challenges include sourcing some of that information if it’s not forthcoming, finding conflicting advice and knowing whether information is ultimately acted upon. Those working in team environments spoke of the value of complementary knowledge/skills and close collaboration (e.g., between the physician and pharmacist). Processes to glean information from other health care providers to inform medication decision-making and processes to inform others about medication changes were described. Such communication can aid patient care through consistent messaging but conversely can be detrimental if messaging differs. One physician spoke about the difficulty in trusting the level of accountability of other health care providers who were not physicians but playing expanded roles in medication management. Opportunities to formalize communication and collaboration processes would be beneficial. The data in this section applies to the overall medication management approach rather than a focus only on prescribing cascades. |
| View of RxCascades (positive and negative views of RxC (RP) collapsed): participants’ negative view towards concept of prescribing cascades – participants’ negative views towards their own suspected/identified prescribing cascades; participants’ positive attitudes towards concept of prescribing cascades – participants’ positive views towards their own suspected/identified prescribing cascades | Some patients feel that it’s useful to have a medication that treats the side effect of another medication. This may be more pronounced when the first medication is being used to treat severe pain; people may be more likely to put up with side effects until they become severe. |
| Inappropriate (HCP): HCP experiences of, attitudes towards, potentially inappropriate prescribing cascades both generally and in particular cases – approximate patient equivalent is ‘negative views of RxC’ | This set of data is also quite small. The notion of “appropriate” vs “inappropriate” depends on the viewpoint of the interviewee, may shift over time and given that it’s often based on an individual assessment of benefit/risk, may not be dichotomous. Health care providers may not think a prescribing cascade exists if they deem it to be “appropriate.” One health care provider identified a list of prescribing cascades that might always be inappropriate, particularly in the context of adverse effects caused by the second drug. |
| Appropriate: HCP experiences of, attitudes towards, potentially appropriate prescribing cascades both generally and in particular cases – patient/resident equivalent falls under ‘positive views of RxC” | Prescribing cascades seem to be considered appropriate when they are historically common or when there is clear benefit to the first drug |
| Willingness to manage RxC: patient’s willingness to maintain a suspected/identified prescribing cascade in the context of managing their symptoms, achieving health priorities – for example, a patient may be experiencing a cascade but does not have an issue with maintaining the status quo because their symptoms are being managed to their satisfaction | This is a small set of coded material. In general, people noted satisfaction with (or were resigned to) the status quo either because they felt they were doing well, or they wanted to follow their physician-recommended course of treatment or they felt they could manage side effects of a second drug because the first drug was controlling more bothersome symptoms. |
| Risk/benefit analysis (HCP): HCP experiences of weighing potential risks and benefits of maintaining a suspected/identified prescription cascade for a given patient | The notion of balancing benefit and risk is inherent to viewing a prescribing cascade as ‘good’ or ‘bad’. The assessment of the balance of benefit/risk can shift over time, affected by the patient’s ongoing experience, age, self-reflection and health care provider uncertainty over whether to make a change in medications. |
| Process of resolving: descriptions of how an identified cascade has been/may be resolved – challenges described in resolving an identified cascade or in resolving cascades in general | The process of ‘resolving’ a prescribing cascade often includes investigation into whether a prescribing cascade indeed exists. It’s difficult to separate the ‘identification’ from the ‘resolving’. A more accurate term may be ‘investigation and management’.  Gradual dose reduction of a potentially causative medication, along with monitoring, is a common strategy used to determine if the medication is contributing to a sign or symptom. Another option is a ‘pause and monitor’ approach in which a medication is stopped for a short period of time (e.g., 2 weeks) to determine if it’s contributing to a sign or symptom. This is then followed by gradual reduction of the second medication to see if it’s still required. When there are several relevant prescribing cascades (e.g., where more than one drug might be contributing to a sign or symptom, or where there multiple different prescribing cascades), there is a need to prioritize which potentially causative drugs to reduce first, consider risk-benefit of each drug, goals of care, the possibility of needing to increase or add a different medication to achieve the goals, and the need for step-wise approach with monitoring for resolution or worsening of symptoms.  This process requires conversations with and agreement from patients and amongst health care providers. Decisions can be influenced by patient priorities (e.g., intolerable symptoms, willingness to change) and by physicians’ assessment of both benefit and risk of the various medications. These decisions and conversations can be more challenging when there is cognitive impairment or limited understanding of medications, or when patients are resistant to changes. Gaining buy-in from patients involves being honest but not alarmist about side effects and potential risks, and appealing to the idea of reduced pill burden. The process is facilitated by regular contact with patients, especially for monitoring (e.g., in a clinic environment, in the home by visiting healthcare providers). Communication with other health care providers is important – both to gather information accurate medication lists, to consult perspectives about benefit/risk of each medication, to obtain consent on changes and to inform about planned changes.  It can be difficult to tell if a prescribing cascade has been ‘resolved’ when there are potentially multiple contributing drugs. Through the process, it is also possible that one might discover that a medication(s) was not the cause of a sign/symptom. “and towards the end of the admission, I sort of came to the conclusion that maybe there’s something else underlying these sweats”. And, in other cases, there may be decision to continue with a prescribing cascade because the benefit of the first drug justifies the addition of the second drug or because side effects from the first drug (even if stopped) are not reversible (prednisone/bisophosphonate example). There are also situations in which a condition may have developed regardless of the drug contributing (e.g., steroid-induced osteoporosis).  Patients (and other health care providers?) do not always consider non-prescription drugs as possible causes (or treatments) of prescribing cascades and this complicates management. For example, caffeine can contribute to insomnia, anxiety, tremor and hypertension. The stopping of caffeine can reduce the need to treat these other conditions with medication. |
| Process of resolving: outcomes: results of attempts to resolve cascades; includes both successful and unsuccessful attempts | There is less data in this coding summary than in others. However, there are several examples of the clinical impact of managing prescribing cascades and its’ resulting improvement in quality of life, which may be a result of reducing or stopping medications but may also be impacted by the interprofessional care (e.g., within the Geriatric Day Hospital) and non-pharmacologic interventions. |
| Impact of RxCascades: codes for patient or HCP stating whether or not the prescribing cascade had any impact on them – patient or HCP descriptions of HOW a cascade has had an impact/WHAT the impact is/suspected to be | There is less data in this coding summary than in others. However, the functional and quality of life impact of the adverse effects of medication are compelling and there are some rich quotes. One patient, in particular (and their family member) spoke about the negative impact of diarrhea likely arising from medication use. A healthcare provider for another patient also commented on the inability of the patient to leave the house without taking loperamide to control diarrhea which was felt to be medication-induced. |
| ***Medication Management***  Contains codes covering aspects of medication management from both R/P and HCP participants. Distinguished from Medication Knowledge by active nature of codes: they describe feelings, impacts, activities/actions, and other attitudes, circumstances, etc which influence how participants approach and engage with medications, both those specific to the case being discussed and with medications/the use of medications in general | The sub-theme around medication management elucidates the beliefs and feelings associated with benefits in continuing current medications; ongoing benefits; and drug effectiveness. Patients preferences and how it impacts medication management emerged within this sub-theme with a focus on preferences, in some cases articulated in their goals of care, or in conversation with patients and health care providers around medication treatment options [i.e., reducing medication to improve blood pressure]; symptom management [i.e., reducing medication to reduce edema or sweating, making patient comfortable], and exploring and using alternative non-pharmacological approaches [i.e., compression stockings] to alleviate symptoms and side effects. Uncertainty around ascertaining prescribing cascades also emerged, particularly with patients where there was a lack of historical timeline of when medications were ordered and when they were on several drugs with multiple prescribers. |
| Alternatives: Use of alternative treatment options (i.e., non-pharmaceutical) in addressing symptoms and/or side-effects in the context of a suspected/identified cascade - For example, opting for a ginger supplement instead of Gravol or cannabis rather than opioids | [none] |
| Belief in benefits: Patient’s expressions of belief that they experience benefits from taking a particular medication - e.g., “I’m not sure what medications do but I feel better when I take them so they must be doing something good” - this may be treated as the ‘subjective’ partner of ‘Drug effectiveness’ | This code includes the perceived beliefs around the benefits that patients and their caregivers associated with taking their medication. Beliefs included wanting the medications to stay the same as they are “doing well”, “satisfied with everything”, and trusted their doctor and not wanting to stop or change their medication[s] as their symptoms would get worse [i.e., “because it is doing good right now … for my swelling”]. For one patient, the thought of stopping or changing medications [i.e., laxative and pain] would “be terrible”. This code also included the health care providers views on the patients being “resistant”; “reluctant”; and “rigid” to adjust their medications [i.e., in one case patient was described as “vigilant and not open to altering the pain medication profile”]. This resistance and reluctance in patients rendered the health care providers as having “a greater hesitancy in making too many adjustment” and view that adjusting medications would create “a lot of anxiety” for the patients.  There was also reference to physicians being reluctant to stop a medication that the patient has been happy with for a long time or what was labelled “inheriting physician” viewing the medication to be doing “something really important”; feeling “conflicted” and not wanting to “rock the boat” if the patient is stable and not deteriorating. |
| Deprescribing: Knowledge and experiences of deprescribing medications particularly in the context of a suspected/identified cascade | This code is closely aligned with the benefits and drug effectiveness codes and includes the knowledge and experiences associated with deprescribing and/or reducing the dose of certain medications that patients are taking within the context of a suspected and/or identified cascade. For some patients, the health care provider described the desire to adjust medications to reduce pill burden [i.e., when patient is decompensating and viewed as being “overmedicated”]; increase function; and mitigate symptoms [i.e., increased sleep, swelling] and the need to balance the function and symptoms.  Within this code, the process of discussing deprescribing and reducing the dose of medications was provided with specific examples [i.e., reduce Trazodone, Baclofen] that involved reducing the dose over time. For the most part, patients were open to reducing medications, particularly when they experience relief of symptoms and side effects, but were less knowledgable about potential and/or actual prescribing cascades. Health care providers also discussed challenges in identifying what was a prescribing cascade related to not knowing the medication history of when and why medications were prescribed. |
| Drug effectiveness: Experiences of effectiveness (i.e., in addressing target symptoms) of particular drugs in context of suspected/identified prescribing cascade e.g., “I know Drug B is working because I haven’t had the symptom Drug B is meant to address since I started taking it”. As well, evidence of effectiveness – this may be treated as the ‘objective’ partner of ‘Belief in benefits’ | This code is closely aligned with the beliefs/benefits code and expands more around the desire to remain on medications that patients, caregivers perceive to be effective in treating their condition [i.e., high blood pressure], disease and symptoms [i.e., I know Drug B is working because I haven’t had the symptom Drug B is meant to address since I started taking it]. Patients described their medications as “working very good”, “pain is well controlled”, “getting relief pretty soon after” on one hand and having fear and anxiety that changing or reducing medications would be less effective on the other hand. People also expressed negative feelings toward their medications. In some cases, patients reduced or stopped taking the medication due to believing the medication was not effective in treating the symptoms [i.e., Diclofenac for pain]. |
| Feelings towards meds (RP): Patient’s feelings towards their own medications as well as towards the idea of taking medications in general. May be positive, negative or neutral. Distinguished from ‘Patient Preferences’ in focus on feelings; for example, a patient may prefer not to be on so many medications but feel positively about their own medications for a given reason | This code is closely aligned with the beliefs/benefits code, drug effectiveness code and delineates patients’ feelings towards their own medications and medications in general. Patients described feeling “very thankful”, being “happy with all my medications”, “good” on one hand and “suspicious” on the other hand. |
| Goals of Care: Describes patient and HCP goals in medication management, treatment choices, etc. Distinguished from ‘Impact of patient priorities’ in that priorities relate to how goals are determined and/or achieved; e.g., a patient may prioritize not having disruptive side effects over potential longevity and the goal becomes to maximize patient comfort | This code includes the goals of care determined by the health care provider/team and goals of care identified/informed by the patient around medication treatment [i.e., reducing medication to improve blood pressure] and symptom management [i.e., reducing medication to reduce edema or sweating, making patient comfortable]. In some cases, patients were involved in setting their goals of care around reducing medications including which medication and timing of reduction; one patient stated wanting to “be able to go out and walk with my walking group” in addition to “no longer be short of breath”. In other cases, patients did not feel they were asked about their goals. |
| Impact of past experiences: Instances where participant’s past experiences with medication, healthcare providers, medical treatment, etc. have, or are suggested to have, an impact on R/P and/or HCP willingness to make changes to medications | This code includes not wanting to adjust medications based on past experiences of adjusting medications and the impact on symptoms [i.e., challenge urinating when Furosemide was reduced]. |
| Impact of patient priorities on HCP decisions: Impact of resident/patient preferences regarding their medications and treatment plans on HCP decision making, particularly with regards to making changes to medications | This code is closely aligned with goals of care code and reflects how patients’ preferences regarding their medications and treatment plans impacts health care providers decision making associated with adjusting and making changes to medications [i.e., making no changes, reducing and/or stopping] due to the goal of alleviating symptoms and side effects associated [perceived or real] with medication [i.e., edema, sweating, dry mouth, constipation] or change in status [i.e., weight gain, decline in cognition]. |
| Initiative and control: Incidents of patient exerting control or taking initiative over their treatment particularly with regards to their medications. May also code for attitudes towards exerting control or taking initiative. For example, a patient who seeks treatment alternatives, proactively addresses their symptoms (e.g., adding supplements, monitoring blood sugar) | This code is closely aligned with goals of care, impact of patient priorities on HCP decisions and includes situations where patients would exert control or take initiative to manage their treatment particularly with regards to their medications. In most situations, patients would either taper/reduce or stop their medication completely due to side effects [i.e., memory problems] and symptoms [i.e., feeling loopy from trazodone] they were experiencing or concern about being on the medication too long. Part of managing their medications included accessing information by researching the medication or consulting with a pharmacist. One patient was described by a health care provider as the “quarterback or the case manager of her own care”. |
| Non-adherence: Describes instances in which a patient is/did not take their medications as indicated by an HCP. For example, a patient relating that they did not want to take a medication anymore and so stopped doing so without discussion with an HCP | This code is aligned with beliefs/benefits code and describes situations where patients did not take their medications as indicated [i.e., stopped taking or opted not to take medication, missed medication], and in some situations did not consult with a health care provider. |
| Patient preferences: Patient’s preferences regarding taking specific medications, i.e., their current or past medications. Patient’s preference regarding taking medications in general; e.g., their preference for not taking medications at all or for seeking/using non-pharmaceutical treatment options, their preference for certain types of medication/treatment over others (e.g., taking insulin instead of medications, taking pills instead of needles). Distinguished from ‘Feelings towards meds’ | This code is closely aligned with benefits/beliefs, feeling towards medications, goals of care, and impact of patient priorities in health care providers and includes the patient’s preferences associated with the medications they are currently are, in the past were, taking. These preferences ranged from wanting the medication(s) to “stay the same”; take less medication(s); and reduce the medication(s). There were also generic references by health care providers around patients being “happy to take less medications” and patients not wanting “to go on any more pills” and trying alternative non-pharmacological options and less invasive administration of medications [i.e., taking pills instead of needles]. |
| Temporal challenges: Challenges described in relating a given emergent symptom/side effect/sign to specific medication(s) due to the time elapsed between starting the medication(s) and emergence of symptom/side effect/sign. May also code for descriptions of the general ease or difficulty experienced in making these links where a significant amount of time, or what the subject views as a significant amount to time, has passed between starting a given medication and the emergence of symptoms/side effects/signs | This code includes the challenges around understanding the “timeline” or “chronology” of when medications were provided to patients and lacking a “historical context”. Without an understanding of the timing, it was difficult to ascertain if a prescribing cascade had occurred/is occurring with the elapse of time between starting of the medication{s) and the emergence of a symptom or side effect. Being on medications for a long period of time was also brought up by both health care providers and patients as a challenge to understanding if symptom and/or side effect was association with the long-standing medication(s). |
| Understanding on-going benefit: Challenges describe in determining whether a given medication remains beneficial in addressing a target symptom/condition/etc. Challenges described in understanding on-going benefit, or lack thereof, of a current medication - for example, a patient has been taking a given medication for many years and it is no longer clear what, if any, benefit they are deriving from it. This patient may have trouble understanding or accepting that they may no longer need such a medication | This code reflects when patients and health care providers were uncertain of the benefits of continuing on the medication(s) and the impact on symptoms [i.e., rage, furosemide] and in one situation that a medication [i.e., atorvastatin] may be contributing to side effects amongst several medications. |
